# Physiological and Pathological Factors Affecting Drug Delivery to the Brain by Nanoparticles

**DOI:** 10.1002/advs.202002085

**Published:** 2021-03-15

**Authors:** Yamir Islam, Andrew G. Leach, Jayden Smith, Stefano Pluchino, Christopher R. Coxon, Muttuswamy Sivakumaran, James Downing, Amos A. Fatokun, Meritxell Teixidò, Touraj Ehtezazi

**Affiliations:** ^1^ School of Pharmacy and Biomolecular Sciences Liverpool John Moores University Byrom Street Liverpool L3 3AF UK; ^2^ Division of Pharmacy and Optometry The University of Manchester Stopford Building, Oxford Road Manchester M13 9PT UK; ^3^ Cambridge Innovation Technologies Consulting (CITC) Limited St. John's Innovation Centre Cowley Road Cambridge CB4 0WS UK; ^4^ Department of Clinical Neurosciences Clifford Allbutt Building – Cambridge Biosciences Campus and NIHR Biomedical Research Centre University of Cambridge Hills Road Cambridge CB2 0HA UK; ^5^ School of Engineering and Physical Sciences Heriot‐Watt University William Perkin Building Edinburgh EH14 4AS UK; ^6^ Department of Haematology Peterborough City Hospital Edith Cavell Campus Bretton Gate Peterborough Peterborough PE3 9GZ UK; ^7^ Institute for Research in Biomedicine (IRB Barcelona) Barcelona Institute of Science and Technology (BIST) Baldiri Reixac 10 Barcelona 08028 Spain

**Keywords:** aging brain, blood–brain barrier model, complement activation, drug delivery to the brain, immunogenicity, nanoparticles, neurodegenerative diseases

## Abstract

The prevalence of neurological/neurodegenerative diseases, such as Alzheimer's disease is known to be increasing due to an aging population and is anticipated to further grow in the decades ahead. The treatment of brain diseases is challenging partly due to the inaccessibility of therapeutic agents to the brain. An increasingly important observation is that the physiology of the brain alters during many brain diseases, and aging adds even more to the complexity of the disease. There is a notion that the permeability of the blood–brain barrier (BBB) increases with aging or disease, however, the body has a defense mechanism that still retains the separation of the brain from harmful chemicals in the blood. This makes drug delivery to the diseased brain, even more challenging and complex task. Here, the physiological changes to the diseased brain and aged brain are covered in the context of drug delivery to the brain using nanoparticles. Also, recent and novel approaches are discussed for the delivery of therapeutic agents to the diseased brain using nanoparticle based or magnetic resonance imaging guided systems. Furthermore, the complement activation, toxicity, and immunogenicity of brain targeting nanoparticles as well as novel in vitro BBB models are discussed.

## Graphical abstract

The treatment of brain diseases is challenging partly due to the inaccessibility of therapeutic agents to the brain. The physiology of the brain alters during neurological diseases, making treatment challenging. Nanoparticles are novel method of drug delivery, however, an understanding of biological responses such as immunogenicity and toxicity is essential for safe application.

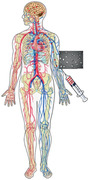

## 1 Introduction

Despite many advances both in understanding and technology, the reliable delivery of treatments to the brain is an unsolved challenge. Nanoparticles (NPs) have gained much recent prominence as a potential tool to achieve this valuable aim.^[^ ^1^, ^2^, ^3^ ^]^ As research in this area has expanded, several of the issues that might increase the likelihood of success or prevent creation of a new therapy have become clearer. In this review, we seek to describe the biological, physiological, and physical background to some of these with the intention of encouraging those working in this area at the same time as providing them with a map that highlights some of the pitfalls that must be avoided.

In terms of opportunities, the latest findings in normal, aged, and diseased brains reveal that in certain disease states and upon aging, changes take place in the blood–brain barrier (BBB) permeability^[^ ^4^, ^5^, ^6^, ^7^, ^8^, ^9^, ^10^, ^11^ ^]^ that could improve the ability of NPs to access the brain.^[^ ^12^ ^]^ These changes impose restrictions on the NPs or nanocarriers (NCs) that differ from those required to access the brain when it is in a normal, healthy state. As a result, researchers have investigated methods to maintain or recover the BBB functionality such as the intravenous (i.v.) injection of mesenchymal stem cells (to inhibit the deterioration of BBB function),^[^ ^7^ ^]^ oral administration of terflunomide (promoting pericyte coverage, pericyte survival and downregulating tight junction degradation),^[^ ^8^ ^]^ or use of *Panax notoginseng* saponins (by activation of Nrf2 antioxidant defense system).^[^ ^13^ ^]^ These approaches could improve the efficacy of drug delivery to the brain by NPs or NCs by restoring the BBB integrity. Furthermore, the receptors involved in the normal functioning of the BBB may be dysfunctional in brain diseases.^[^ ^14^, ^15^ ^]^ Finally, the physiological environment of the brain may change due to chemicals or diseases.^[^ ^16^, ^17^, ^18^ ^]^

In this review article, the details of the healthy state are first described and then the variations in a variety of states of disease, damage or aging. Technological innovations have provided researchers with new tools to study the BBB and these are beginning to also include variations that reflect the changes in the BBB in disease states but gaps are highlighted by our comprehensive survey such as models for the aging BBB.

In terms of challenges, NPs have been found to have a particular ability to provoke complement activation that can lead to severe immune responses; this too is dependent upon the characteristics of the NPs.^[^ ^19^, ^20^, ^21^ ^]^ The ability of NPs to cause cytotoxicity^[^ ^22^ ^]^ via either apoptosis^[^ ^23^, ^24^ ^]^ or necrosis^[^ ^25^ ^]^ are explored as are further aspects of immunogenicity,^[^ ^26^ ^]^ hemolytic properties^[^ ^27^ ^]^ and more general toxicity.

Throughout the review, detailed tables provide information about the behavior of a variety of NPs of different composition and morphology that highlight the breadth of research in this area and provide design guidelines that will enable studies to focus on NPs with an increased likelihood of becoming part of new therapeutic modalities.

## 2 Physiological Barriers to the Brain Parenchyma

There are five barriers between the brain and peripheral tissues in adults:^[^ ^28^ ^]^

- The BBB, at the capillaries of the brain parenchyma
- The arachnoid barrier at the meninges
- The blood cerebrospinal fluid (CSF) barrier covered by the epithelial cells at the choroid plexus
- The circumventricular organs (CVOs) barrier formed by tight junctions between adjacent tanycytes
- The glia limitans interface between the CSF and the brain parenchyma.

These are explained in the following.

### 2.1 Blood–Brain Barrier

**Figure 1:**
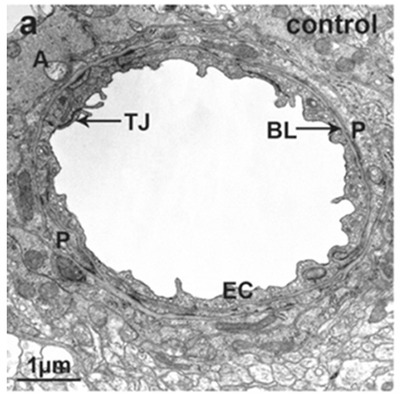
TEM image of healthy human neurovascular unit in the brain. Reproduced with permission.^[^ ^30^ ^]^ Copyright 2011, IntechOpen. (P: pericyte, BL: basal lamina, EC: endothelial cell, A: astrocyte, TJ: tight junction.

The BBB maintains a homeostasis within the brain environment, which is essential to the normal functions of neurons in the brain. The BBB achieves this partly by shielding the neural cells from harmful agents in the blood, but this protective role of the BBB makes drug delivery to the brain more challenging compared to other organs, where blood capillary fenestration facilitates delivery of the drug molecules to the target cells. To overcome the BBB, NPs and NCs have been developed, which cross the BBB by mechanisms such as receptor mediated transcytosis (RMT), a process inherent to normal functioning brain endothelial cells.^[^ ^1^, ^2^, ^3^ ^]^ **Figure** **1** presents a transmission electron microscopy (TEM) image of a normal human neurovascular unit. The tight junction (TJ) is presented as an electron‐dense elongated line, with the basal lamina (BL) encompassing an endothelial cell (EC), surrounded by pericytes, but with gaps in the BL, with astrocyte end‐feet located behind. Neuropils can also be identified. The central nervous system (CNS) contains both micro‐ and macrovessels. These occupy 25–30% of the total brain volume. The total surface area of microvasculature is 12 m^2^ in the adult brain, which corresponds to ≈100 cm^2^ g^−1^ of brain tissue. The total length of capillaries in the brain is about 650 km. These capillaries are typically 40 µm apart and the capillary lumen diameter is about 6 µm. TJs are formed between two overlapping separate impermeable cell membranes, one on the inside of the vessel wall (luminal side) and the other on the outside (abluminal side). Hence, the TJ is a slant cut rather than a straight short cut from the luminal side to the abluminal side. Typically the gap is between 300 and 500 nm between the luminal side and abluminal side of brain microvessels.^[^ ^29^ ^]^

**Figure 2:**
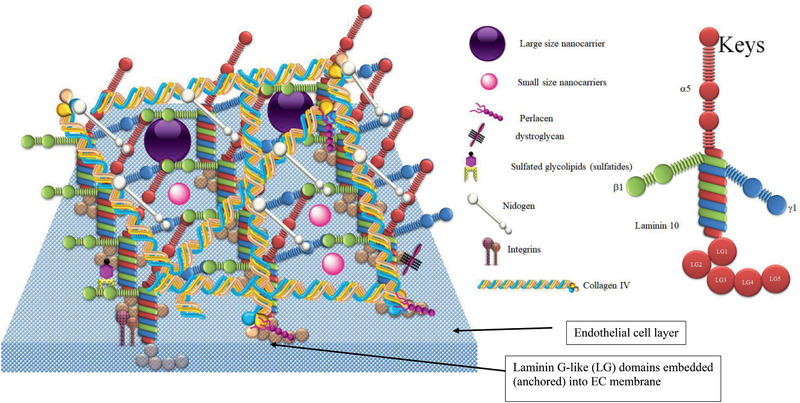
Schematic presentation of basal lamina (BL) in the neural vascular unit (NVU). This diagram presents a protein network within the BL, which affects the diffusion of NCs that cross the BBB toward brain parenchyma, with smaller NCs being more efficient in crossing the network compared to larger NCs, which may be trapped in the protein network.

In the young rat brain, the thickness of the basal lamina (also known as basement membrane, BM) is 50 nm in the frontal cortex and 49 nm in the hippocampus CA1 region.^[^ ^31^ ^]^ The BL is composed of three layers. Layer one, produced by EC, contains laminin‐8^[^ ^32^ ^]^ and laminin‐10.^[^ ^32^, ^33^ ^]^ The middle layer contains collagen IV,^[^ ^34^, ^35^ ^]^ agrin,^[^ ^36^ ^]^ elastin by pericytes,^[^ ^37^ ^]^ perlecan,^[^ ^38^, ^39^ ^]^ and fibronectin.^[^ ^34^, ^35^ ^]^ Layer three contributed by astrocytes contains laminin‐1 and laminin‐2.^[^ ^32^ ^]^ Nidogen‐1 is spread within the BL and links collagen IV to laminin to form a 3D matrix (**Figure** **2**),^[^ ^40^ ^]^ lack of nidogen‐1 results in discontinuation of BL in the brain capillaries.^[^ ^41^ ^]^ This complexity of BL further restricts the mobility of NPs in the brain, in particular larger NCs (>100 nm) (Figure 2).^[^ ^42^ ^]^ However, it should be noted that the NP size is not the only factor that can block the NP in the BL.^[^ ^43^ ^]^ Adsorption of proteins and surface charge of NPs could play major roles in slowing the NPs diffusing through the BL.^[^ ^43^ ^]^ This could reduce the relocation of the NP within the brain parenchyma, as the BL is continued to the extracellular matrix (ECM).^[^ ^44^ ^]^

Pericytes are completely embedded within the BL. Laminin 8 (containing *α*4 chain) facilitates transmigration of T‐cells from the blood into the BL under inflammatory conditions.^[^ ^32^ ^]^ Activated T‐cells interact with intercellular adhesion molecule 1 on the surface of ECs and can induce a transient breakdown of the barrier.^[^ ^45^ ^]^ Further dislocation of T‐cells into the neural tissue depends on the permission of local macrophages. Local macrophages and ECs will release matrix metalloproteinases (MMP), if relocation of T‐cells is needed to digest ECM and make a path for T‐cells.^[^ ^46^, ^47^ ^]^ Previous studies have shown immune reactions in the brain toward NPs. Therefore, the BL could be the site where NCs are identified as foreign bodies in the brain and cytokines are released.

### 2.2 The Arachnoid Barrier at the Meninges

**Figure 3:**
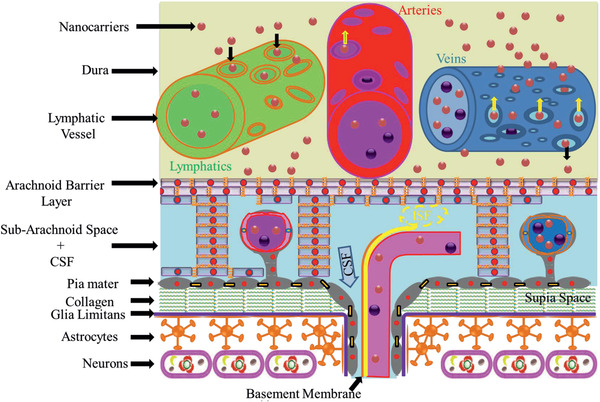
A schematic diagram of arachnoid barrier at the meninges. This diagram shows that NPs may exit the fenestrated blood vessels in the dura mater. It also shows that CSF in the subarachnoid space (SAS) enters the brain parenchyma via the paravascular space and interstitial fluid ISF exits the brain parenchyma along the BL and mixes with CSF in the SAS. The arachnoid barrier separates the dura mater from the SAS, and the pia mater separates brain parenchyma from the SAS. Glia limitans and astrocytes form a barrier between the pia mater and the brain parenchyma. The subpia mater is mainly composed of collagen fibers.

**Figure** **3** presents a schematic diagram of the meninges. The dura mater is about 1 mm thick in the human brain, and it is composed of dense fibrous tissue. Microscopically, the dura consists of densely packed bundles of collagen fibers with interspersed arteries, veins, and lymphatics.^[^ ^28^ ^]^ The dura mater lymphatic vessels contribute to the clearance of macromolecules from the brain.^[^ ^48^ ^]^ The blood vessels are fenestrated in the dura mater.^[^ ^49^ ^]^ It may be, therefore suggested that NCs may leave blood vessels via the fenestrated blood vessels in the dura mater.

This could be an explanation for the weak appearance of nontargeted NPs in the brain as observed in previous studies.^[^ ^50^ ^]^

The arachnoid covers the inner aspect of the dura mater and forms the arachnoid blood‐CSF barrier, separating the CSF in the subarachnoid space (SAS) from fenestrated blood vessels in the dura mater. In humans, the arachnoid barrier is 200 µm thick, and the main body of the arachnoid is composed of closely packed leptomeningeal cells joined by desmosomes and devoid of basement membranes. On the other hand, the arachnoid cells adjacent to the dura are joined by TJs, which prevent solutes or cells gaining access to the SAS or conversely solutes from the SAS to the dura mater.

However, a recent study suggests that drug molecules exiting the fenestrated blood vessels in the dura mater may be transported to the CSF in the SAS by transporters on the arachnoid barrier.^[^ ^51^ ^]^ Similarly, the drug molecules may be transported from the CSF in the SAS back to the dura mater and then into blood capillaries. Two key transporters have been identified in the arachnoid barrier of mouse: 1) P‐glycoprotein (P‐gp), and 2) breast cancer resistance protein (BCRP).^[^ ^51^ ^]^ BCRP and P‐gp are also highly expressed in human arachnoid tissue.^[^ ^51^ ^]^ Sheet‐like and filiform trabeculae with cores of collagen fibers coated by leptomeningeal cells extend from the arachnoid barrier across the human SAS to join the pia on the surface of the brain parenchyma (subarachnoid space plus CSF depicted in Figure 3).

Trabeculae divides the SAS into compartments with suspended arteries and veins within the SAS.^[^ ^52^ ^]^ As shown in Figure 3, there are major arteries in the SAS, which branch perpendicularly into the brain parenchyma.^[^ ^53^ ^]^ In humans, the pia mater is composed of a thin layer of leptomeningeal cells that are joined by gap junctions.^[^ ^54^ ^]^ The pia mater is closer to the astrocytes (glia limitans) but it is separated from these by the subpia mater, which contains blood vessels and collagen.^[^ ^28^, ^55^ ^]^ The pia mater covers the surface of arteries and veins that leave the SAS, cross the subpia mater and enter the brain parenchyma.^[^ ^55^ ^]^ Figure 3 also presents the flow of CSF from the SAS into the paravascular space,^[^ ^56^ ^]^ and the reverse flow of interstitial fluid (ISF) along the BM in the tunica media of cerebral arteries that form the intramural peri‐arterial drainage (IPAD).^[^ ^57^ ^]^ When 15 nm gold NPs were injected into mouse CSF, the NPs appeared in the basement layer of cortical arteries. This shows that cerebral vascular basement membranes form the pathways for fluid passing into and out of the brain.^[^ ^58^ ^]^ Thus tracers or drug molecules present in the CSF will enter the brain alongside arteries and leave the brain back to the CSF, but along separated periarterial BM pathways.^[^ ^57^ ^]^ Alternatively, if NCs in the brain parenchyma release their cargo, the drug molecules will enter the CSF via the convective ISF flow and re‐enter the brain parenchyma via the CSF flow into the paravascular space.

### 2.3 The Blood CSF Barrier at the Choroid Plexus

**Figure 4:**
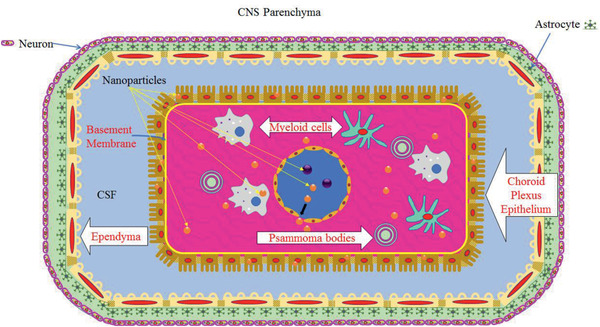
A schematic diagram of choroid plexus in the brain. Choroid plexus is a highly vascularized tissue with fenestrated blood vessels which provides the opportunity for small NCs to exit the blood vessels and enter the tissue of the choroid plexus (stroma). The choroid plexus is located within each ventricle of the brain, and it is separated from the CSF in the ventricles by epithelial cells, which have numerous villi on the surface. As a consequence of aging, calcified bodies are formed within the stroma called psammoma bodies. Brain parenchyma is separated from the CSF by ependymal cells. Also, white blood cells (myeloid cells) exit the blood vessels in the choroid plexus and occupy the stroma.

A schematic diagram of the choroid plexus in the brain is presented in **Figure** **4**. The choroid plexus is a highly vascularized tissue with numerous villi on the surface; it is located within each ventricle of the brain. The choroid plexus is separated from the CSF by single‐layered epithelium cells, and brain parenchyma are separated from the CSF in the ventricles by ependymal cells. The capillaries in the choroid plexus are separated from the epithelial cells by a thin layer of connective tissue called stroma. Bundles of collagen are present in the stroma of the choroid plexus, but they are surrounded by leptomeningeal cells. In older humans, there are spheres of collagen fibers, produced and surrounded by leptomeningeal cell. However, these may become calcified to form calcospherites in the stroma of the choroid plexus (called psammoma bodies).^[^ ^59^, ^60^, ^61^ ^]^

The epithelium cells in the choroid plexus form TJs, however, the electrical resistance (200 Ω cm^2^)^[^ ^62^ ^]^ is estimated to be much less than the BBB (1870 Ω cm^2^).^[^ ^63^ ^]^ This implies that the choroid plexus epithelium membrane is leaky.^[^ ^64^ ^]^ The choroid plexus has been suggested to be a site for entry of pathogens into the brain tissue.^[^ ^65^ ^]^ Pathogens may enter through the transcellular or paracellular routes of epithelial cells in the choroid plexus, or in infected phagocytes via the “Trojan‐horse” mechanism.^[^ ^66^ ^]^ Figure 4 also presents schematically the potential dislocation of NPs from the perforated blood vessels in the choroid plexus into the stroma.^[^ ^67^, ^68^ ^]^ Most likely, the NPs will remain in the stroma due to the TJs at the epithelial cells of the choroid plexus,^[^ ^69^ ^]^ however, they may enter into the brain tissue via the Trojan‐horse mechanism.^[^ ^70^ ^]^ It should be noted that epithelial cells in the choroid plexus may uptake NPs;^[^ ^68^ ^]^ and the epithelial cells of the choroid plexus may transport NCs into the CSF via RMT.^[^ ^71^ ^]^

The choroid plexus could also be a another point of entry for NPs to the brain, when the surface of NPs is decorated with a hybrid ligand (e.g., combination of transferrin receptor (TfR) and cohesin domain from *Clostridium thermocellum*).^[^ ^71^ ^]^ In another study, uncoated poly(butyl cyanoacrylate) (PBCA) NPs (185–200 nm) managed to accumulate in the brain at concentrations below 1% of the dose after i.v. administration in Wistar Unilever rats.^[^ ^72^ ^]^ Although this is not considerable, the NPs might have been accumulated in the choroid plexus and partly entered the brain via the Trojan‐horse mechanism using immune cells. Coating these PBCA NPs with polysorbate 80 (Tween 80) doubled the amounts of NPs accumulated in the brain.^[^ ^72^ ^]^ Moreover, uptake of untargeted‐albumin NPs (208 nm) by the BBB ECs has been observed, but these particles were not seen in the brain parenchyma after i.v. injection into SV 129 mice.^[^ ^73^ ^]^

On the other hand, apolipoprotein E (ApoE) coated albumin NPs (249 nm) were found in all brain regions and neurons.^[^ ^73^ ^]^ It is unlikely that ApoE NPs would be able to access all the brain regions solely by crossing the BBB, due to their large sizes. Most likely, they also crossed epithelium cells of the choroid plexus,^[^ ^74^ ^]^ which led to the distribution of NPs in other regions of the brain via the CSF.

Hence, the size and surface decoration of NCs play important roles in targeting different regions of the brain.

### 2.4 Circumventricular Organs Barrier

CVOs are highly vascularized brain structures with fenestrated blood vessels and neurons. CVOs permit sensing of hormones in the blood and the release of hormones to the blood. These structures allow the brain to monitor the blood without compromising the BBB. There are three sensory CVOs: the subfornical organ, the organum vasculosum of the lamina terminalis, and the area postrema. These CVOs permit neurons to sense the blood, and relay related information to other regions of the brain. Also, there are four secretory CVOs: the neurohypophysis, the median eminence (ME), the intermediate lobe of pituitary gland, and the pineal gland.^[^ ^75^ ^]^ The subcommisural organ is an indiscriminate CVO, which means that some classify it as a CVO,^[^ ^76^ ^]^ whereas other do not.^[^ ^75^ ^]^ Indeed, one reason that it might not be considered as a CVO is because this organ lacks fenestrated capillaries.^[^ ^77^ ^]^

Ependymal cells border CVOs. These form the lining of both ventricles and the brain parenchyma side, which is bordered by astroglial cells.^[^ ^78^ ^]^ All three TJ proteins, ZO‐1, occludin and claudin1, appeared at the lining of ventricles of mouse brain,^[^ ^78^ ^]^ with occludin and ZO‐1 appearing on the brain parenchyma side.^[^ ^79^ ^]^ These proteins create a barrier around CVOs such that staining molecules for example Evans blue (MW = 960.81 Da) remain within CVOs following i.v. injection.^[^ ^78^, ^79^ ^]^

**Figure 5:**
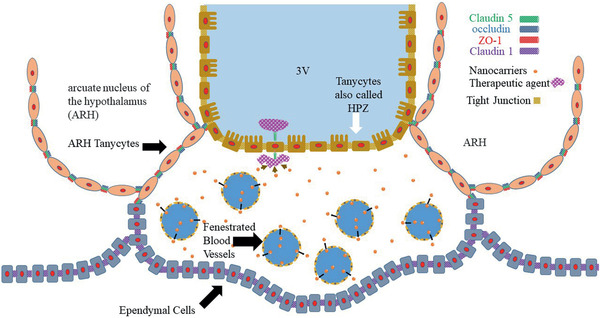
Schematic diagram of the median eminence (ME), one of the circumventricular organs (CVOs). This diagram shows three types of ependymal cells: the ventral part (also called HPZ cells), the arcuate nucleus of the hypothalamus (ARH), and the border (the bottom part). This figure shows occludin and ZO‐1 at the ARH ependymal cells, all three TJ proteins (ZO‐1, occluding, and claudin1) at the ventral side, and claudin 1 at the border side. The ME contains rich fenestrated capillaries, which could allow dislocation of NCs from the blood vessels into the CVOs. The tanycytes with their TJ proteins prevent diffusion of NCs to the brain parenchyma. However, the drug molecules released from NCs may enter the CSF in the ventricle by the transporter/receptors at the HPZ cells.

A schematic diagram of the ME is illustrated in **Figure** **5**. This figure shows three types of ependymal cells: the ventral part, the arcuate nucleus of the hypothalamus (ARH), and the border (the bottom part). Figure 5 shows occludin and ZO‐1 at the ARH ependymal cells, all three TJ proteins (ZO‐1, occludin, and claudin1) at the ventral side, and claudin 1 at the border side. The ME contains rich fenestrated capillaries, which could allow translocation of NCs from the blood vessels into the CVOs. This is also shown schematically in Figure 5. Experimental evidence suggests that the ependymal cells (at the ventral border) can transport macromolecules such as leptin^[^ ^80^ ^]^ into the blood via transcytosis from the CSF^[^ ^81^ ^]^ and vice versa.

Therefore, if NCs release drug molecules, they may be transported across the ependymal cells into the CSF (shown schematically in Figure 5).

### 2.5 Glia Limitans

**Figure 6:**
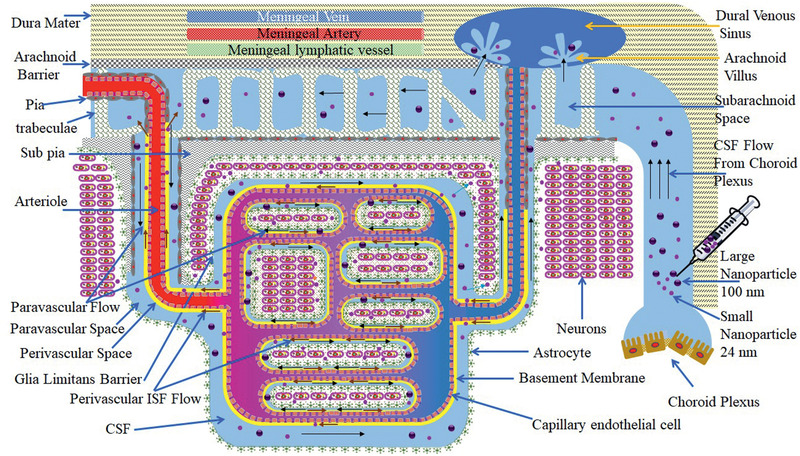
Schematic presentation of the glia limitans interface between the CSF and the brain parenchyma and the circulation of the CSF in the brain. This diagram presents injection of NCs/NPs with two sizes into the CSF large and small. Large NCs/NPs will circulate within the brain as well as the small NCs/NPs but will not cross the glia limitans, while small NCs/NPs will cross the glia limitans and penetrate into the brain parenchyma. This figure also shows that NCs/NPs will start their journeys from the ventricle (site of injection) and via the CSF will flow into the SAS, and then into the brain through the perivascular space along the arteries. After transporting along the BL and along the walls of veins, the NCs/NPs will return into the SAS. Due to the size of NCs/NPs (both large and small sizes), they will not be able to leave the CSF via the arachnoid villus into the dural venous sinus, which joins the systemic circulation.

The glia limitans is a CNS barrier formed by astrocyte endfeet that protects the brain parenchyma. **Figure** **6** presents a schematic diagram of the glia limitans barrier in the brain. Glia limitans forms around blood vessels, hence NPs or inflammatory cells that cross the BBB will face this barrier. Glia limitans also forms beneath the pia mater, and hence protects the brain from inflammatory cells or NPs in the CSF. It should be noted that glia limitans is permissive to molecules in a size dependent manner. Tracers with molecular weights less than 2000 kDa managed to cross the glia limitans, and molecules with sizes of 759 Da easily crossed the glia limitans and were distributed widely in the brain parenchyma following injections into the cisterna magna.^[^ ^56^ ^]^ Therefore, glia limitans acts like a sieve to protect the brain parenchyma from harmful molecules. In addition, IgG molecules (≈10 nm in size) could not cross the glia limitans following injection into the cisterna magna.^[^ ^82^ ^]^

On the other hand, NPs with a size of 24 nm crossed the glia limitans following convection‐enhanced delivery to the cortex of rats.^[^ ^83^ ^]^ This technique involves the infusion of NP solution into the brain parenchyma, with transport of NPs into the brain interstitium being driven by a pressure gradient. However, 100 nm NPs remained in the arteriol perivascular space, and could not cross the glia limitans even by applying this technique.^[^ ^83^ ^]^

These studies suggest that NCs should have sizes in the range of 10–24 nm to cross the glia limitans and penetrate into the brain parenchyma following crossing the BBB or after intrathecal injection. It should be added that larger NPs may manage to cross the glia limitans, if the surface of the NPs is decorated with appropriate targeting ligands. For example, as explained above, albumin NPs decorated with ApoE entered neurons following i.v. injection into mice.^[^ ^73^ ^]^ The NPs had an average diameter of 249 nm. However, the number of these NPs in the neurons was scarce, suggesting the prevention of NP transport into the brain parenchyma by the glia limitans. By referring to the use of exosomes in drug delivery to the brain,^[^ ^84^ ^]^ it may be accepted that the NPs/NCs should have surface decoration with brain targeting ligands and sizes preferably less than 80 nm, or ideally around 24 nm. To further support this claim, adeno associated viruses (AAVs, 20–25 nm) have been considered for delivery for DNA to the brain following i.v. injection.^[^ ^85^, ^86^, ^87^ ^]^

The CSF is produced mainly by the choroid plexus, and this is schematically shown in Figure 6. The rate of right lateral ventricular CSF formation was determined in the range of 0.0622 to 0.103 mL min^−1^,^[^ ^88^ ^]^ while another work found that the rate of CSF formation was in the range of 0.083 to 0.103 mL min^−1^ in three adult patients (two with meningeal cancer and one with dementia).^[^ ^89^ ^]^ CSF flows from the choroid plexus to the SAS and part of it enters the brain via the paravascular space surrounding the descending arterioles to the brain. CSF circulates back to the SAS via the perivascular space around the cerebral veins. Part of the CSF leaves the SAS toward dural venous sinus through the arachnoid villus.

Figure 6 also presents distribution of large (100 nm) and small (24 nm) NCs in the brain following intrathecal injection. The basis for these choices is according to previous investigations. The crossing of 24 nm NPs through the glia limitans has been shown, but 100 nm NPs remain within the perivascular space.^[^ ^83^ ^]^ This figure presents that small NCs would cross the glia limitans and enter the brain parenchyma, but larger NPs will be circulating in the CSF. These NPs will not be able to leave the CSF via the arachnoid villus, nor crossing the arachnoid barrier and entering dura mater unless they can be transported back to the blood by transcytosis via the transporters in the arachnoid barrier or the BBB, or taken up by macrophages in the brain.

**Figure 7:**
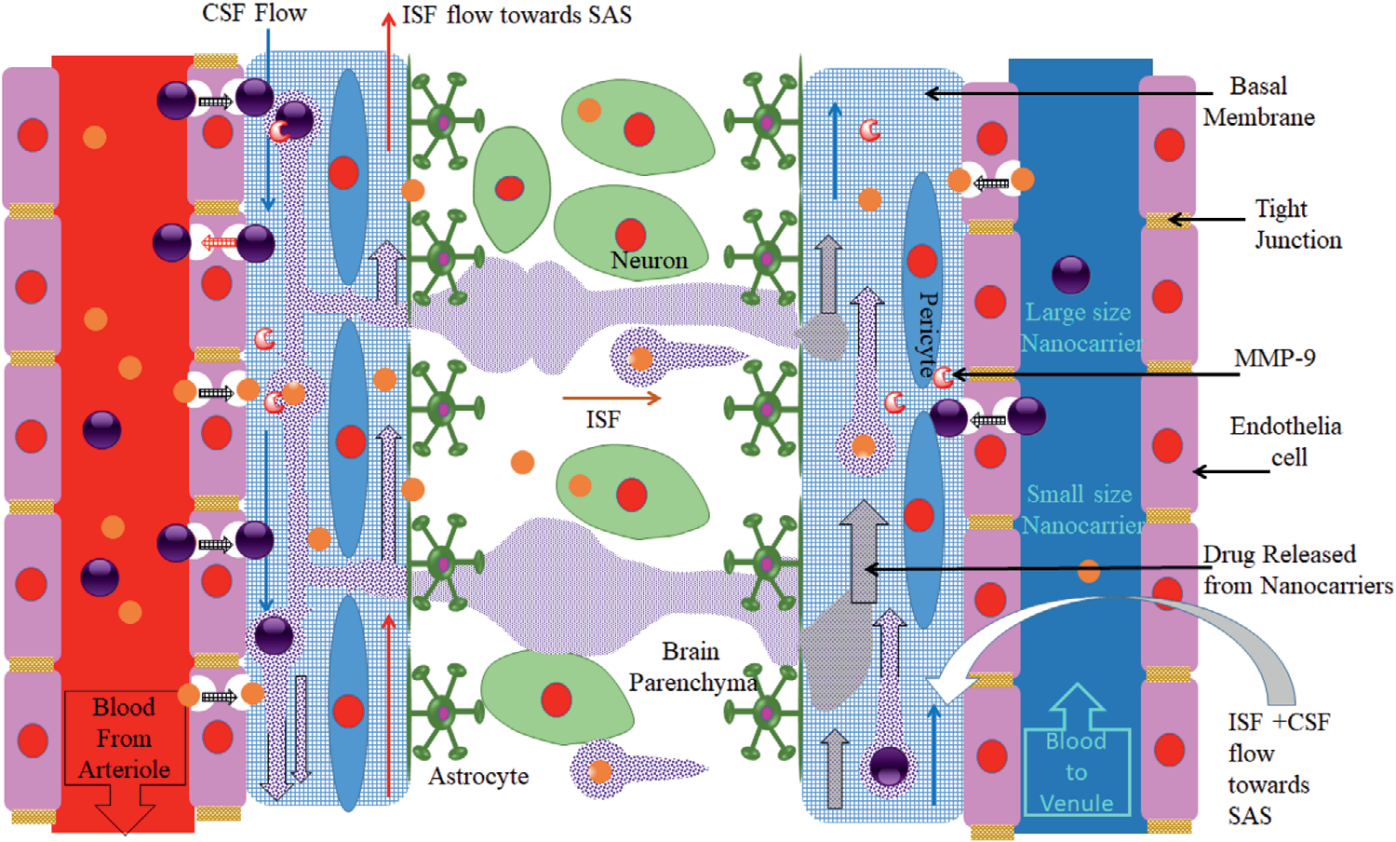
A schematic depiction of the glymphatic pathway. This diagram shows that CSF arrives in the brain through paravascular paths, by passing the glia limitans, washes the brain parenchyma, mixes with ISF, leaves the brain parenchyma through the opposite‐side glia limitans, and is cleared from the brain via paravenous paths. In this diagram, two different sizes of NPs are shown (small and large). Large NPs will stay within the arteriole paravascular path, while small NPs will cross the glia limitans and reach the brain parenchyma. The small NPs may leave the brain by following the convective flow of ISF. In addition, large NCs may release their cargo in the basement membrane due to degradation by enzymes such as MMP‐9. The cargo would be distributed within the brain by the convective flow of the ISF in the brain.

A schematic depiction of the glymphatic pathway is shown in **Figure** **7**. CSF enters the brain through the paravascular paths, crosses the glia limitans, sweeps the brain parenchyma, mixes with ISF, leaves brain parenchyma through the opposite‐side glia limitans, and is cleared from the brain via paravenous paths. In this diagram, NPs are shown with two different sizes (small and large). Large NPs (100–200 nm) will remain within the arteriole paravascular path, while small NPs (24 nm) will cross the glia limitans and reach the brain parenchyma. The small NPs would leave the brain by following the convective flow of ISF. As explained in the previous sections, the relocation of NPs in brain parenchyma is not only size dependent. The interaction of NPs with extracellular matrix, and surfaces of the cells will affect their distribution in the brain.

Figure 7 also presents that large NCs may release their payload in the BM due to degradation by enzymes such as MMP‐9. Hence, the payload may be transported to the brain parenchyma by the convective flow of CSF to the brain parenchyma. It is also suggested that ISF flows in the opposite direction to the CSF toward arteriole paravascular space and eventually into the CSF in the SAS.^[^ ^28^ ^]^ Therefore, part of the released cargo might be transported toward CSF in the SAS and distributed in a wider area of the brain.

The above sections show the barriers that NPs or NCs face when these cross the BBB, and also other potential barriers that NPs/NCs may utilize to access the brain parenchyma. In the above sections emphasis is given to particle size, although this is a major factor, other properties of NPs or NCs such as charge should be take into consideration for diffusion of NPs/NCs in the brain parenchyma.

Mechanisms are described in the above sections for crossing the NPs through the CNS barriers based on published physiological literature. However, further studies are required to add more details about the interaction of NPs with the CNS. Although animal models make valuable contribution to understanding drug delivery to the brain by NPs, it remains difficult to conduct mechanistic studies on the barrier function and interactions with NPs. Therefore, a microphysiological platform of the BBB was engineered to better understand the transport of NPs through the BBB. For example, this model allowed authors to determine that apolipoprotein A1‐based NPs (eHNP‐A1) crossed the BBB through the scavenger receptor class B type 1 (SR‐B1) via transcytosis. Blocking SR‐B1 reduced the transport of the NPs through the BBB, however, the NPs still crossed the BBB via alternative transport mechanisms. Therefore, the use of eHNP‐A1 was suggested for drug delivery to the brain.^[^ ^90^ ^]^

## 3 Effects of Aging on the BBB

Aging affects both barrier and transport functions of the BBB. In terms of barrier functionality, by aging, the human BBB permeability increases, in particular to albumin,^[^ ^91^ ^]^ with human albumin having a diameter of 8.5 nm.^[^ ^92^ ^]^ During aging of the human brain, the BBB breakdown begins in the hippocampus, which leads to damage to pericytes.^[^ ^93^ ^]^ However, the BBB permeability increases further in patients with either vascular dementia or AD compared with age‐matched controls.^[^ ^91^ ^]^ There are several factors that contribute to the BBB breakdown by aging. For example, acid sphingomyelinase (ASM) is a critical factor in the integrity of the BBB and ASM levels increase with aging in the endothelial cells of human brain, leading to greater BBB permeability through increased caveolae‐mediated transcytosis.^[^ ^94^ ^]^ Furthermore, by aging microglia are primed to present amplified responses to immune challenges.^[^ ^95^ ^]^ This is partly due to the presence of dystrophic microglia (loss of fine branches) in aged humans.^[^ ^96^ ^]^ Activated microglia disrupt the BBB.^[^ ^97^ ^]^

The BBB exchange and transport functionality also change with aging. For example, it has been found that in adult rats the transport of anti‐TfR antibody (OX26) was decreased with respect to the brain of younger rats following i.v. administration of OX26.^[^ ^98^ ^]^ As another example, the insulin receptor density decreases with aging in human brain.^[^ ^99^ ^]^ Insulin‐receptor exhibiting cells are present on the luminal membrane of the BBB and neural cells in the CNS,^[^ ^100^, ^101^ ^]^ which import blood‐borne insulin into the brain via RMT.^[^ ^100^ ^]^ Efforts to exploit potential use of insulin for drug delivery via RMT has stalled due to a short serum half‐life of about 10 min and the possibility of hypoglycaemia (too much insulin will promote widespread uptake of glucose from the blood circulation).^[^ ^102^ ^]^ This problem may be overcome by employing insulin‐like growth factors (IGF‐1 and IGF‐2),^[^ ^103^ ^]^ as higher concentrations of IGFs are required to develop hypoglycaemia.^[^ ^102^ ^]^ Nevertheless, antibody meditated binding to insulin receptor has been exploited for drug delivery to the brain.^[^ ^104^ ^]^ Boado and Pardridge fused a lysosomal enzyme, *α*‐l‐liduronidase (IDUA), with monoclonal antibodies to human insulin receptor (HIRMAb) to cross the BBB. Upon administration in rhesus monkeys, 1.2% of injected drug accumulated in the brain, whereas the unconjugated IDUA did not penetrate the BBB.^[^ ^105^ ^]^ It is reported that HIRMAb acts like a molecular Trojan horse to deliver the IDUA across the BBB by binding to insulin receptors. Furthermore, they demonstrated the delivery of a decoy receptor (tumor necrosis factor (TNF) receptor) across the BBB by fusing it with HIRMAb.^[^ ^106^ ^]^

The expression of low density lipoprotein receptor‐related protein 1 (LRP‐1) also decreases on the BBB upon aging.^[^ ^107^ ^]^ This could affect uptake of NPs that target LRP‐1. Therefore, targeting other receptors as well as LRP‐1^[^ ^108^ ^]^ could be a suitable strategy for drug delivery to the aged brain by NPs. Likewise, the activity of P‐gp decreases on the human BBB with aging.^[^ ^109^ ^]^ NP formulations have been developed to silence P‐gp on the BBB.^[^ ^110^ ^]^ This was to improve the delivery of drugs to the brain by preventing efflux of drug molecules or NPs taken up by the BBB endothelial cells back to the blood (Figure 7, return of NP is indicated by red arrow). However, it should be noted that lower expression of P‐gp may increase accumulation of AD‐associated amyloid beta (A*β*) deposits in the brain.^[^ ^111^ ^]^

There are also morphological changes to the brain blood vessels associated with aging. The tortuosity of cortical arteries increased in aging mice. In addition, older mice had uneven distribution of capillary vessels in the brain, signs of vascular structure modification with aging.^[^ ^112^ ^]^ Similarly, microvascular density of the paraventricular nucleus reduced in humans upon aging.^[^ ^113^ ^]^ Astrocyte and pericyte number increase (20% and 22%, respectively) in the cortex of rat brains during aging.^[^ ^114^ ^]^ Furthermore, collagen deposits form around the brain blood vessels during aging,^[^ ^115^ ^]^ a phenomenon known as microvascular fibrosis.^[^ ^116^ ^]^ This could be due to the reduction of the cellular vascular reactivity (CVR) induced by aging (evident in the hippocampus of aging subjects).^[^ ^117^ ^]^ CVR is a brain blood vessel response to external vasoactive stimuli in the hippocampus, which contributes to memory.^[^ ^117^ ^]^ In addition, microvasculature in the brain become thickened mostly due to hyalinization.^[^ ^118^ ^]^ There is an 18% increase in the diameter in the frontal cortex BM (from 50 to 59 nm) and 32% increase in diameter of hippocampal BM (from 49 to 65 nm) in the rat brain upon aging.^[^ ^31^ ^]^ Notably, the BM thickness was almost doubled in aged mice.^[^ ^119^ ^]^Similarly, it has been shown that BM thickness typically increases in humans throughout aging from 70 nm at fetal age to about 3500 nm (3.5 µm) at age 90.^[^ ^115^ ^]^ These morphological changes to the brain microvascular structure would affect the efficiency of NPs reaching the brain parenchyma and neurons compared to brain in younger ages.

Aging not only brings increased BBB permeability, but also this involves neuroinflammation as the result of cytokine release such as interleukin (IL)‐1*β* and interferon (INF)‐*γ*.^[^ ^120^ ^]^ It is believed that such neuroinflammation played a major role in preventing BBB transmigration of inhaled 21.5 nm TiO_2_ NPs.^[^ ^120^ ^]^ It should be noted that in this study aging caused increased BBB permeability to small molecules such as atenolol, but not NPs.^[^ ^120^ ^]^ In addition, aging was associated with a dramatic decline in the efficiency of exchange between the SAS CSF and the brain parenchyma.^[^ ^121^ ^]^ This could lead to impaired glymphatic flow in the brain, in particular in the BM of the BBB, which could affect distribution of NPs that crossed the BBB. Lack of NP clearance from the BM of the BBB may increase the chance of returning of the NPs back to the blood via transcytosis.

In summary, the brain microvessels are changed with aging, and although they become more permeable, deposition of collagen, albumin, micro‐hemorrhages, thickened BM, and neuroinflammation do not make the barrier easier to bypass by NPs. It may be suggested that small NPs (perhaps less than 20 nm) would have a better chance to cross the BBB, bypass the deposited serum proteins around brain microvessels and reach the brain parenchyma in aged subjects.

## 4 Effects of Disease on the BBB

Several diseases affect the BBB function in various aspects. However, there is a compensatory mechanism in the brain that protects neurons from exposure to toxic chemicals in the serum. In the following sections, the effects of brain diseases on the BBB are discussed, and the consequences are evaluated for targeting of the brain by NPs.

### 4.1 Alzheimer's Disease

The neural vascular unit (NVU) is affected by Alzheimer’s disease (AD) in several different ways. The deposition of A*β* in the brain is the hallmark of AD, with A*β* plaques depositing around brain vessels.^[^ ^122^ ^]^ This brings structural damages to the NVU including the BBB wall and surrounding extracellular space. In addition, amyloid fibrils were observed in small leptomeningeal arteries and perforating cortical arterioles of patients with AD.^[^ ^123^ ^]^ The deposits were typically between 8 and 10 nm^[^ ^123^ ^]^ and affect pericytes, which maintain integrity of the BBB.^[^ ^124^ ^]^ Accelerated pericyte degeneration and a reduction in their number have been shown in AD.^[^ ^125^, ^126^, ^127^ ^]^ Furthermore, perivascular microglial proliferation was prominent in the hippocampus of patients with AD;^[^ ^127^ ^]^ and A*β* deposits caused astrocyte endfeet swelling and retraction in transgenic arcA*β* mice.^[^ ^128^ ^]^

The interaction of A*β* plaques with the receptor for advanced glycation end products (RAGE) reduced the expression of TJ proteins.^[^ ^129^ ^]^ In addition, the A*β* oligomer itself increased the expression of RAGE, which further reduced the levels of ZO‐1, claudin‐5 and occludin.^[^ ^130^ ^]^ Accordingly, the TJ proteins (claudin‐5, ZO‐1 and occludin) were lost in the brain tissues of patients with capillary cerebral amyloid angiopathy (CAA),^[^ ^131^ ^]^ as A*β* deposits were found in the cerebral veins of the majority (78%) of patients with CAA.^[^ ^132^ ^]^ These observations show that A*β* plaques affect both morphology and function of the BBB.

Aquaporins are protein channels involved in water transport. Aquaporin 4 (AQP4) is involved mainly in interstitial brain fluid homeostasis, including BBB regulation. The expression of AQP4 was significantly higher in AD patients compared to control subjects.^[^ ^133^ ^]^ On the other hand, the expression of perivascular AQP4 decreases with increased A*β* deposition.^[^ ^134^ ^]^ These alterations in the expression of AQP4 may contribute to edema formation in the brains of patients with AD and alteration in the BBB permeability. At this stage, it is not clear how these changes in water transport in the AD brain may affect the retention time of NPs.

In humans, ApoE has three isoforms: ApoE2, ApoE3, and ApoE4. Carriers of ApoE4 have a major risk factor for AD. The carriers of ApoE4 show greater breakdown of BBB by age compared to ApoE2 or ApoE3 alleles, which leads to considerable albumin levels in the CSF.^[^ ^135^ ^]^ Furthermore, brain samples from AD cases homozygous for ApoE *ε*4 showed increased deposition of fibrin(ogen) specifically in CAA and oligomeric A*β*‐positive vessels compared with AD ApoE *ε*2 and *ε*3 allele carriers.^[^ ^136^ ^]^

The presence of A*β* plaques and elevated RAGE expression enhanced MMP secretion from the brain capillaries in an animal model of AD.^[^ ^129^ ^]^ Also A*β*_1‐42_ (as oligo) increased the levels of MMP‐2 and MMP‐9 in the brain ECs.^[^ ^130^ ^]^ Furthermore, MMP‐9 is also accumulated in pericytes of AD ApoE4 carrier patients.^[^ ^125^ ^]^ These enzymes further affect the BBB integrity and make it more permeable. On the other hand, the presence of these enzymes could open an opportunity for the development of enzyme‐responsive NCs for drug delivery to the brain.^[^ ^137^ ^]^

Recent studies identified that serum levels of bile acids increased in patients with AD compared to control subjects.^[^ ^138^, ^139^, ^140^ ^]^ Bile acids (deoxycholic acid and chenodeoxycholic acid) increased permeability of the BBB by activation of Rac1 followed by phosphorylation of occludin, with chenodeoxycholic acid being more potent.^[^ ^141^, ^142^ ^]^ The disruption of the BBB occurs more with hydrophobic bile acids than hydrophilic bile acids (such as ursodeoxycholic acid).^[^ ^141^ ^]^ The disruption of the BBB could lead to microhemorrhages with the release of neurotoxic hemoglobin‐derived products.^[^ ^143^ ^]^

As the results of the above findings, the two‐hit‐vascular hypothesis has been proposed for developing AD.^[^ ^144^ ^]^ The hypothesis states that first (hit 1) the cerebrovascular vessels get damaged. This is sufficient to initiate neurodegeneration. In addition, and perhaps as a result, A*β* plaque accumulation (hit 2) further contributes to the BBB damage, and these contribute to developing AD.^[^ ^144^ ^]^ Therefore, the increased BBB permeability would be the start of AD development. It has been suggested that the delivery of genes to the brain ECs might restore LRP‐1 levels at the BBB. This is to contribute the clearance of A*β* from the brain and minimizing BBB damage.^[^ ^144^ ^]^

On the other hand, the expressions of ECM proteins (collagen IV, perlecan, fibronectin) increase in the frontal and temporal cortex of patients with AD.^[^ ^145^ ^]^ The capillary BM thickness increased significantly in the hippocampus, cerebral cortex and thalamus of patients with AD.^[^ ^116^, ^146^ ^]^ This was accompanied by increased expression of BM proteins (collagen IV, laminin, and nidogen‐2).^[^ ^146^ ^]^ Unfortunately, this alteration of the BM would reduce the drainage of A*β* from the brain.^[^ ^146^ ^]^ It is shown that fibrin deposition increases in the AD brain, which correlates with the degree of disease pathology.^[^ ^147^ ^]^ Furthermore, brain microbleeds have been shown by using magnetic resonance imaging (MRI) in preclinical AD patients, which further contributes to the deposition of serum proteins around brain microvessels.^[^ ^148^ ^]^ In severe AD, the shrinkage of ECs has been observed.^[^ ^149^ ^]^ In addition, expression of glucose transporters at the BBB, which mediate glucose transport into the brain, decreases in AD leading to increased BBB leakage.^[^ ^150^ ^]^ The increased permeability of the BBB in AD leads to the deposition of albumin,^[^ ^93^, ^151^ ^]^ fibrinogen,^[^ ^152^ ^]^ fibrin,^[^ ^126^, ^150^ ^]^ IgG,^[^ ^126^, ^150^ ^]^ and prothrombin^[^ ^149^ ^]^ in microvascular segments in particular in those areas that have A*β* deposition.^[^ ^151^, ^153^ ^]^ Furthermore, the cerebral levels of vitamin D binding protein (DBP) increase from 0.6 µg mL^−1^ in control subjects to 1.2 µg mL^−1^ in patients with AD^[^ ^154^ ^]^ to inhibit aggregation of monomeric A*β*_1–42_^[^ ^155^ ^]^ and prevent BBB microbleeds. Therefore, although the permeability of the BBB increases in AD, these compensatory mechanisms would protect the neurons from harmful materials originating from the serum. These compensatory mechanisms may bring further hindrance for NPs to cross the BBB.

**Table** **1** (in vivo) and **Table** **2** (in vitro) present preclinical examples for the development of NP formulations in relation to the treatment of AD. It should be noted that Talamini et al. found that gold NPs as small as 10 nm could not enter the brain parenchyma following i.v. administration to adult male CD‐1 mice.^[^ ^156^ ^]^ Therefore, the penetration of NPs into the brain in the examples below may indicate compromised BBB in animal models of AD.

**Table 1:** Recent NP formulations developed in preclinical studies for the treatment of AD. PLGA: poly(lactic‐*co*‐glycolic acid)

Nanocarrier type	Drug	Targeting ligand	Size [nm]	Delivery route	Model	Remarks	Ref.
Poly(lactic‐co‐glycolic acid) (PLGA)	DBP	None	226.6 ± 44.4	i.v.	5× transgenic mice	Reduced accumulation of A*β* in the brain Reduced the presence of Iba‐1 NPs restored cognitive function	^[^ ^157^ ^]^
Liposomes with cardiolipin	Curcumin/Nerve growth factor (NGF)	Lipid‐conjugated wheat germ agglutinin	135.2 ± 6.8	i.v.	AD rats	Reduced brain A*β* plaques	^[^ ^158^ ^]^
PLGA	Curcumin	Selenium	160 ± 5	i.v.	5XFAD	Penetrating into the brain	^[^ ^159^ ^]^
PLA‐polyethylene glycol (PEG)	Curcumin	None	<80	Oral	Tg2576	Improved cue memory in the contextual fear‐conditioning test	^[^ ^160^ ^]^
PLGA	A*β* generation inhibitor peptide (PQVGHL)	CRTIGPSVC (targets TfR)	139.8	i.v.	Transgenic AD mice	Reducing cognitive impairments, cytokine production, brain ROS, and A*β* levels	^[^ ^161^ ^]^
PLGA	A*β* generation inhibitor peptide (PQVGHL)	None	128.6	i.v.	Transgenic AD mice	Presence of NPs in the brain and reducing cognitive impairments, cytokine production, brain ROS and A*β* levels	^[^ ^161^ ^]^
Multiwalled carbon nanotubes	Berberine and phospholipid	Tween 20	125–295	i.v.	AD rats	Improved behavioral outcomes	^[^ ^162^ ^]^
Dendrigraft poly‐l‐lysines (third generation)	d‐peptide +RNA (BACE1)	RVG	97	i.v.	APP/PS1 transgenic mice	Reducing the formation of A*β* plaques and improving the Morris water maze results	^[^ ^24^ ^]^
Solid Lipid Nanoparticles (SLNP)	Galantamine hydrobromide	None	<100	Oral	AD rats	Improving the Morris water maze results	^[^ ^163^ ^]^
Amphiphilic compound of phenylboronic groups	Curcumin	KLVFFAED (targeting RAGE)	65	i.v.	APP/PS1 mice	Significantly improved memory behavior	^[^ ^164^ ^]^
Selenium	None	CGHKAKGPRK	95	–	–	Reduced A*β* fibrillization inside human brain ECs and PC12 cells	^[^ ^165^, ^166^ ^]^
PEG‐PDMAEMA(poly[(2‐(*N*,*N*‐dimethylamino) ethyl methacrylate])	siRNA (BACE1)	CGN (*d*‐CGNHPHLAKYNGT) + QSH (A*β* targeting ligand)	70	i.v.	APP/PS1 mice	Downregulating BACE1 at both mRNA and protein levels	^[^ ^167^ ^]^
PEG‐gold	Anthocyanins	None	135 ± 5	i.v.	AD mice	Reduced A*β*_1–42_ induced memory deficits, the levels of A*β*, BACE‐1, and APP	^[^ ^168^ ^]^
Liposomes	Plasmid DNA	Dual targeting 1) Transferrin protein+ 2) penetratin (RQIKIWFQNRRMKWKK)	150.4 ± 3.75	i.v.	C57BL/6J mice	12% of the administered dose were found per gram of brain tissue	^[^ ^169^ ^]^
Liposomes	Plasmid DNA	None	NA	i.v.	C57BL/6J mice	8% of the administered dose were found per gram of brain tissue	^[^ ^169^ ^]^
Liposomes	Rivastigmine	Sodium taurocholate	340 ± 10	i.p.^a)^	Balb‐C type mice	Decreased acetylcholinesterase activity	^[^ ^170^ ^]^
Liposomes	Phosphatidic acid/cardiolipin	None	102 ± 2	i.p.	APP/PS1	Reduced the levels of A*β* both in serum and the brain	^[^ ^171^, ^172^ ^]^
Chitosan	Piperine	None	248.50 ± 23.50	Nasal	AD mice	Improved cognitive function	^[^ ^173^ ^]^

^a)^ Intraperitoneally.

**Table 2:** Recent in vitro studies of developing NPs for the treatment of AD. Tf: transferrin; TfR: transferrin receptor

Nanocarrier type	Drug	Targeting ligand	Size [nm]	Remarks	Ref.
SLNP	Resveratrol/grape‐skin or grape‐seed extracts	OX26 mAb^a)^	168–189	Reduce A*β*_1‐42_ aggregation NPs crossed in vitro BBB model	^[^ ^178^ ^]^
SLNP	Rapamycin (an mTORC1 inhibitor)	Tween 80	70–750	Higher encapsulation efficiency with Compritol	^[^ ^179^ ^]^
PLGA	iA*β*_5_ (LPFFD)^b)^	OX26 + anti‐A*β* (DE2B4) antibody	166 ± 2	The cell uptake NPs increased from 8% (with OX26 only) to 14% (with DE2B4 and OX26) by porcine BCECs	^[^ ^180^, ^181^ ^]^
Gold	None	None	30	Inhibiting A*β* fibrillization	^[^ ^182^ ^]^
PEG‐SPIONS^c)^, ^d)^	None	None	20	Retarded fibrillization of A*β*	^[^ ^183^ ^]^

^a)^ Anti‐Tf receptor monoclonal antibody

^b)^ Inhibitor of A*β* formation

^c)^ Superparamagnetic iron oxide nanoparticles

^d)^ Superparamagnetic iron oxide nanoparticles (SPIONS) were withdrawn from the market due to safety issues.^[^ ^184^ ^]^

The results in Table 1 suggest that NP sizes both above and below 100 nm have been effective in ameliorating memory deficits and brain A*β* levels in the AD animal models. However, it appears that sub 100 nm NPs are more potent. The research on animal models of AD suggest a considerably compromised BBB, perhaps partly by A*β*‐oligomer disrupted blood‐CSF barrier in the choroid plexus,^[^ ^174^ ^]^ which may allow the NPs to enter into the brain parenchyma following exiting from the blood through fenestrated blood vessels in the choroid plexus. It should be noted that NPs may interact with A*β* in choroid plexus,^[^ ^175^ ^]^ and exert their therapeutic effects. Hence, the clinical outcomes were similar for both types of NPs (with or without brain targeting ligand).^[^ ^161^ ^]^ Nevertheless, NPs with small sizes (70 nm) with brain targeting ligands could invoke superior therapeutic effects due to crossing the BL, glia limitans and distribution in brain parenchyma.^[^ ^176^ ^]^ It should be noted that expression of TfR decreases in the hippocampus of patients with AD compared to age‐matched controls.^[^ ^177^ ^]^ Therefore, NP uptake may be affected in AD when NPs utilize transferrin mediated transport across the BBB.

In terms of using NCs for the treatment of patients with AD, CERE has been developed, which is an AAV serotype 2 vector expressing human NGF. These are delivered to the brains of patients with AD by injection into the brain (a single stereotactic neurosurgical procedure under general anesthesia). CERE‐110 passed phase I clinical testing,^[^ ^185^, ^186^ ^]^ and has proceeded to phase II clinical trial with 49 AD patients. Although AAV2‐NGF delivery was well‐tolerated, it did not affect clinical outcomes nor did it modulate selected AD biomarkers. However, in the treatment group, the mini‐mental state exam of some patients increased four points.^[^ ^187^ ^]^ It should be noted that pre‐existing anti‐AAV antibodies prove to be an obstacle in the use of AAVs in gene delivery to the brain,^[^ ^85^, ^188^ ^]^ which could necessitate the administration of AAVs by intracerebral injections to avoid potential interaction with pre‐existing antibodies in the blood.

As explained above, there are several mechanisms that increase permeability of the BBB in patients with AD compared to age‐matched control subjects.^[^ ^91^ ^]^ These observations lead to the conclusion that although the BBB may become leaky in AD (animal models), and this would help the NPs crossing the BBB, the diffusion of NPs may be reduced in the brain due to A*β* deposits and the extra residues of proteins in the ECM leaked from the serum as compensatory mechanism to work against the BBB disruption.^[^ ^116^ ^]^

Despite extensive research into novel therapies for AD, the drug development has proven to be unusually difficult with a 99.6% failure rate in the decade of 2002 to 2019.^[^ ^189^, ^190^ ^]^ Past clinical trials can provide lessons to apply to future trials and drug development for AD treatment. One of the lessons that can be related to NPs is: Ensuring that the drug within the NP enters the brain and it is not removed from the brain by transporters such as P‐gp.^[^ ^191^ ^]^

### 4.2 Multiple Sclerosis

Dysfunction of the BBB is also a major hallmark of multiple sclerosis (MS).^[^ ^192^ ^]^ However, the BBB integrity appears to be much higher in patients with MS compared to experimental autoimmune encephalomyelitis (EAE) mice. There is an extensive change to the BL in the inflammatory regions of MS lesions. The BL becomes irregular and discontinuous.^[^ ^193^, ^194^ ^]^ Niche‐like perivascular areas form in EAE mice that can accommodate neural stem cells (NSCs) to promote brain repair.^[^ ^195^ ^]^ Dysfunction of the BBB is also a major hallmark of MS.^[^ ^192^ ^]^ TJ protein expression was decreased in the BCECs, and the loss of ZO‐1 was prominent in patients with MS.^[^ ^192^, ^196^ ^]^ The disruption allows the leakage of fibrinogen from the blood into the CNS,^[^ ^192^ ^]^ and this can be an activator of microglia.^[^ ^192^ ^]^ Exposure of ECs to pro‐inflammatory cytokines (IFN‐ *γ*, TNF‐*α*, IL‐1*β*) interrupts the BBB by decreasing endothelial expression of occludin (disorganizing cell‐cell junctions),^[^ ^197^ ^]^ losing claudin‐3 from TJs,^[^ ^198^ ^]^ decreasing expression of ZO‐1,^[^ ^196^ ^]^ and promoting the shedding of endothelial “microparticles.”^[^ ^199^ ^]^ The active lesions showed TJ abnormalities in the brain of patients with MS, and TJ abnormality was associated with the leakage of serum protein (fibrinogen).^[^ ^200^ ^]^ The hydrodynamic diameter of fibrinogen is about 22 nm.^[^ ^201^ ^]^ Despite this observation, the levels of fibrinogen in the CSF of healthy subjects have been measured to be slightly higher (3.84 µg mL^−1^) than CSF levels of patients with MS (< 2 µg mL^−1^).^[^ ^202^, ^203^ ^]^ These observations indicate again that NCs/NPs should be less than 20 nm to cross the BBB in patients with MS due to its dysfunction. Larger NCs/NPs will require crossing the BBB endothelial cells via transcytosis.

Neuropilin 1 (NRP1) is highly expressed in the brain endothelial cells of patients with MS, in particular in the early active demyelinating lesions;^[^ ^204^ ^]^ and the interaction of IFN‐*γ* and NRP1 may contribute to the dysfunction of the BBB in MS.^[^ ^204^ ^]^ Furthermore, secreted protein acidic and rich in cysteine (SPARC) is a cell‐matrix modulating protein that is involved in endothelial barrier function. SPARC reduced ZO‐1 and occludin expression in a model of the BBB, and promoted the permeability of the BBB in a concentration dependent manner.^[^ ^196^ ^]^ As cerebral blood vessels become intensely SPARC positive in EAE mice, then SPARC would further contribute to enhanced BBB permeability in MS.^[^ ^196^ ^]^

Peripheral blood lymphocytes have greater ability to degrade laminin in MS patients compared to normal controls.^[^ ^205^ ^]^ In MS, the breakdown of the BBB facilitates leukocyte transmigration to the lesions via recognition of vascular adhesion protein 1.^[^ ^206^ ^]^ Furthermore, SPARC may promote transmigration of leukocytes across the BBB.^[^ ^196^ ^]^ In addition, the serum levels of MMP‐9 were increased in patients with MS and this could contribute to the breakdown of the BBB.^[^ ^207^ ^]^ To maintain the integrity of the BBB, MS patients present elevated hedgehog signaling components in the brain^[^ ^208^ ^]^ and the astrocyte‐secreted Sonic hedgehog is essential for the integrity of the BBB.^[^ ^208^ ^]^ The presence of leukocytes around the BBB may increase up take of NPs that crossed the BBB, hence, reducing the number of NPs reaching the brain parenchyma.

The leakage of the BBB in MS suggests that NPs would reach the neurons in the brain more effectively compared to the healthy BBB following i.v. administration. In the following, examples are provided. NPs of PLGA with a size of 217 nm were injected intravenously into EAE mice and small amounts of NPs were subsequently found in the brain. It was suggested that the NPs penetrated into the brain via a leaky BBB and choroid plexus in EAE mice.^[^ ^209^ ^]^ In another study, nanoliposomes with a size of 80 nm penetrated into the brain tissue of EAE mice 3–6 fold more than control (normal) mice following i.v. administration. It should be noted that the amount of liposomes in the brain was about 3% of injected dose per mg of brain tissue.^[^ ^210^ ^]^ Nanoliposomes with an average size of 74 nm had the same therapeutic effects as targeted‐nanoliposomes to the brain in EAE mice.^[^ ^211^ ^]^ Nontargeted nanoliposomes (nanosterically stabilized liposomesn (SSL); 80 nm in diameter) contained the prodrug prednisolone and showed a five‐fold higher therapeutic efficacy than the free drug. This was attributed to the accumulation of nSSL in the brain due to compromised BBB.^[^ ^212^ ^]^ Cerium oxide NPs (2.9 nm diameter) also penetrated into the brain of EAE mice.^[^ ^213^ ^]^ In addition, very small superparamagnetic iron oxide particles (VSOPs) with a hydrodynamic diameter of 7 nm penetrated into the perivascular inflammatory lesions of EAE mice following i.v. administration.^[^ ^69^ ^]^ VSOPs were also observed in the choroid plexus.^[^ ^69^ ^]^ In addition, pomegranate seed oil nanodroplets had diameters of 30 and 180 nm, but the larger size nanodroplets were more effective in the treatment of EAE mice following oral administration.^[^ ^214^ ^]^ Finally, curcumin dendrosomes decreased the scores of disease in EAE mice following intraperitoneal (i.p.) administration;^[^ ^215^ ^]^ and the effects of curcumin dendrosomes (size of 142 nm^[^ ^216^ ^]^) were attributed to its antiinflammatory effects by regulation of T helper 2 cytokines.^[^ ^215^ ^]^ These studies suggest that in mouse model of MS, NCs in a wide size range of 2.9–217 nm can penetrate into the brain tissue, due to the leaky BBB.

The NP accumulation profile changes in the brain of patients with MS, compared to animal models of MS. Ultrasmall superparamagnetic iron oxide NPs (USPIOs) with size of 20–40 nm diameter^[^ ^217^, ^218^ ^]^ showed a lower abundance in the brain lesions of patients with MS compared to gadolinium‐diethylenetriaminepentaacetic acid (DTPA) (MW = 938 Da).^[^ ^219^ ^]^ It was suggested that USPIOs accumulated inside phagocytic cells. Hence, USPIOs showed infiltration of macrophages into the brain.^[^ ^219^ ^]^ This could indicate that in MS patients, the permeability of the BBB increases only for small molecules such as gadolinium‐DTPA, but less for small NPs such as USPIOs. This could be due to the leakage of serum proteins such as fibrinogen^[^ ^200^ ^]^ into the BL and creating a secondary barrier.

### 4.3 Ischemic Stroke

Ischemic stroke is the reduction of cerebral blood flow to the brain due to the obstruction of arteries, usually by blood clots. Ischemic stroke can cause death or disability. In ischemic stroke the levels of MMP‐2 are known to increase in the ischemic core of nonhuman primates,^[^ ^220^ ^]^ which contributes to the degradation of the BL. In addition, the levels of MMP‐9 were found to be increased in the ischemic brains of nonhuman primates, leading to intracerebral hemorrhage.^[^ ^221^ ^]^ It is estimated that the BBB opening occurs with an average of 6.8 h after onset of ischemia in humans.^[^ ^222^ ^]^ This situation may hint at a delivery window for brain‐targeting NCs/NPs in the early hours of the stroke, also after reperfusion, when there is a greater chance of BBB disruption.^[^ ^222^ ^]^ The BBB disruption is associated with intracerebral hemorrhage (hemorrhage transformation) and poor clinical outcomes.^[^ ^222^ ^]^ These studies suggest that MMP inhibitors would reduce neuronal damage and subsequent disability in ischemic stroke, as shown in animal studies.^[^ ^223^ ^]^ Following stroke, there is an excessive water accumulation in the BL.^[^ ^224^ ^]^ Also it has been shown that the degradation of BL proteins occurs following subarachnoid hemorrhage and cerebral ischemia.^[^ ^225^, ^226^ ^]^

**Figure 8:**
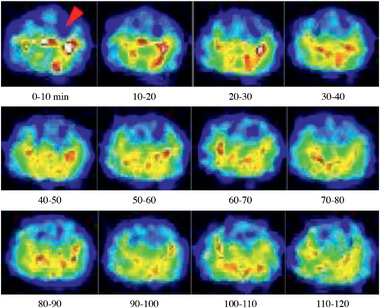
PET imaging of [^18^F]‐labeled PEG‐liposomes in the ischemic region of p‐MCAO rats. p‐MCAO rats were injected intravenously with [^18^F]‐labeled PEG‐liposomes at 1 h after the onset of occlusion. The distribution of [^18^F] Step 2 was determined for 2 h with the Clairvivo PET system. Each single image for every 10 min period was obtained by integration of the total photon numbers during this period. The arrow indicates the ischemic region, and the right hemisphere of these images shows the ischemic side. Reproduced with permission.^[^ ^228^ ^]^ Copyright 2014, International Center for Artificial Organs and Transplantation and Wiley Periodicals, LLC.

A question may be raised that in a brain ischemic stroke, when the blood vessels are blocked, how can NCs get access to the ischemic areas? It has been shown that 100 nm liposomes accumulate in the ischemic core and penumbra region when they are intravenously injected into a permanent middle cerebral artery occlusion (p‐MCAO) rat model despite a significant reduction in the cerebral blood flow.^[^ ^227^ ^]^ Positron emission tomography (PET) showed 100 nm ^18^F labeled PEG liposomes accumulating in the ischemic core; but they started accumulating in the penumbra region first and then gradually moved toward the ischemic core.^[^ ^228^ ^]^ It should be noted that liposomes were not tagged with brain‐targeting ligands, and the accumulation was due to enhanced permeability and retention (EPR).^[^ ^228^ ^]^ **Figure** **8** shows the PET imaging of ^18^F labeled PEG liposomes in the ischemic region of p‐MCAO rats.^[^ ^228^ ^]^ The red arrow indicates the ischemic region. It can be seen from this figure that liposomes were spread in the brain, apart from the ischemic core. However, the liposomes started filling the surrounding region of the ischemic core and gradually progressing toward the center. The presence of the liposomes in the brain would be due to crossing of the disrupted BBB at the penumbra region. Also, this study would suggest the ISF flow around the core, but impaired at the core‐center,^[^ ^229^ ^]^ which leads to gradual progression of the liposomes to this area. It has been shown that the ischemic stroke impairs ISF drainage, in particular along occluded vessels.^[^ ^229^ ^]^

In a recent study, block copolymers were produced containing either NH_2_‐norleucine‐TPRSFL‐C‐SH, a thrombin‐cleavable peptide (T), or NH_2_‐LGRMGLPGK‐C‐SH (M), a MMP‐9‐cleavable peptide.^[^ ^230^ ^]^ The block copolymers were obtained by conjugating the PEGylated peptides to poly(*ε*‐caprolactone) monomers. PEG‐T‐poly(*ε*‐caprolactone) or PEG‐M‐poly(*ε*‐caprolactone) monomers were used to form NCs by precipitation, and these were cleavable by thrombin, or MMP‐9, respectively.^[^ ^230^ ^]^ The PEG‐T‐poly(*ε*‐caprolactone) NPs expanded from sub 100 nm size to over 1000 nm size after 24 h incubation with thrombin. A similar observation was made for PEG‐M‐poly(*ε*‐caprolactone) NPs in the presence of MMP‐9. The aim of the expanding NCs was to prevent return of NCs from the abluminal side of the BBB to the luminal side by transcytosis. To add therapeutic benefits, the NCs encapsulated glyburide to deliver the drug to the ischemic brain (following i.v. administration), and surface conjugated with plerixafor (AMD3100, an immunostimulant) to target the ischemic region of the brain.^[^ ^230^ ^]^ AMD3100‐conjugated NCs were administered i.v. to transient‐MCAO (t‐MCAO) mice. The NCs demonstrated greater specificity to the ischemic regions, as the levels of the C‐X‐C chemokine receptor type 4 (CXCR4) expression were significantly elevated in the ischemic brain (AMD3100 is an agonist to CXCR4 receptor).^[^ ^230^ ^]^ This is interesting, as in an ischemic brain, the presence of NCs will be required only in the ischemic regions. It was found that the glyburide‐loaded AMD3100‐conjugated NCs improved mouse survival by about 50% over 7 days, reduced infarct volume by 36%, and improved neurological scores on a five point scale from 5 (no spontaneous motor activity) to almost 1 (normal motor function).

In a similar approach, PEGylated lipid NPs were conjugated with Fas ligand antibody (Fas‐PLNPs), and Fas is selectively expressed in brain ischemic region.^[^ ^231^ ^]^ The NPs had a size of 60.97 ± 7.95 nm, while non‐Fas‐antibody conjugated control NPs were smaller (38.23 ± 3.22 nm). Non‐Fas‐antibody conjugated NPs accumulated in the brain following i.v. administration to t‐MCAO mice, but in a wider region not exclusive to the ischemic region. This suggests that NCs crossed the BBB due to increased permeability of the BBB in the ischemic and penumbra regions, but the small size of the NPs allowed spreading in the brain both by diffusion in the brain parenchyma and convection dislocation by the ISF flow in the brain. Exclusive localization of Fas‐antibody conjugated NPs at the ischemic region would suggest NP surface attachment in the ischemic region was the contributing factor.^[^ ^231^ ^]^ The Fas‐PLNPs were loaded with 3‐n‐butylphthalide (a neuroprotective agent in ischemic stroke), and administered i.v. to t‐MCAO mice, which improved clinical scores (reduced infarct area and neurological deficits) compared to administration of the drug alone.^[^ ^231^ ^]^ **Table** **3** presents part of recent work on the treatment of ischemic stroke by NPs. There is a secondary brain damage in cerebral ischemia‐reperfusion injury. Reperfusion is considered to be initial restriction of blood supply followed by subsequent vascular restoration and concomitant reoxygenation of downstream tissue.^[^ ^232^ ^]^

**Table 3:** Summary of recent research work on the delivery of NPs for the treatment of ischemic stroke

Nanocarrier	Drug	targeting Ligand	Size [nm]	Route	Model	Remarks	Ref.
PEG‐liposomes	ZL006, a neuroprotective agent	T7 (HAIYPRH)[brain targeting] + SHp (CLEVSRKNC) [stroke homing]	96.24 ± 1.13	i.v.	MCAO‐rat	Liposomes remained in the ischemic region after 24 h significant decrease of cell death much reduced infarct volume (from 60% to 30%	^[^ ^234^, ^235^ ^]^
PLGA (poly(*ε*‐carbobenzoxyl‐l‐lysine)	Lexiscan (modulates the BBB and hence allows more NCs to cross the BBB) + NEP_1‐40_ ‐a 40‐amino acid peptide, an antagonist of Nogo‐66	Chlorotoxin (targeting MMP‐2)	151.8	i.v.	MCAO‐mouse	NPs reduced the infarct area (from 40% to 20%)	^[^ ^236^ ^]^
Liposomes	Fasudil (Rho kinase inhibitor with neuroprotective effects in ischemic stroke)	None	126.8 ± 3.1 nm	i.v.	MCAO‐rat	tPA administered i.v. prior to administration of NPs to activate MM‐2 and MMP‐9 leading to increased BBB permeability Fasudil liposomes significantly suppressed brain cell damage	^[^ ^237^ ^]^
PEG‐liposomes	Asialo‐erythropoietin	None	ND	i.v.	t‐MCAO‐rat	Liposomes prevented brain damages because of reperfusion after ischemic stroke AEPO‐liposomes ameliorated neuronal apoptosis AEPO‐liposomes recovered from the paralysis of the right paw	^[^ ^238^, ^239^ ^]^
Liposomes	Fasudil	None	100	i.v.	t‐MCAO‐rat	Reduced infarct volume (from 0.4 to 0.2 cm^3^) Improved motor function deficits (from motor score 9 to motor score 12 on a 21‐point neurological assessment scale)	^[^ ^240^ ^]^
squalenoyl adenosine	Adenosine	None	120	i.v.	ischemic‐reperfusion rat model	Reduced infarct volume from 54 ± 3 mm^3^ (vehicle treated) to 24 ± 4 mm^3^	^[^ ^241^ ^]^
PLGA‐PEG	Thyroid hormone	Glutathione	326.6	i.v.	MCAO‐mouse	As well as NPs coated with glutathione, uncoated NPs also significantly reduced infarct volume	^[^ ^242^ ^]^
Chitosan	Z‐DEVD‐FMK^a)^	TfR antibody	637 ± 2	i.v.	MCAO‐mouse with reperfusion	NPs reduced infarct volume from 43 ± 4 mm^3^ (control) to 3 ± 2 mm^3^. NPs released sufficient amounts of the active ingredient to prevent activation of caspase 3 following reperfusion.	^[^ ^243^ ^]^

^a)^ *N*‐benzyloxycarbonyl‐Asp(OMe)‐Glu(OMe)‐Val‐Asp(OMe)‐fluoromethyl ketone.

AEPO: Asialo‐erythropoietin

Comparing the above results and findings in Table 3 suggests that the BBB is more compromised in a MCAO‐mouse model compared to a MCAO‐rat model. Hence larger‐untagged NPs (about 300 nm) can cross the BBB in a MCAO‐mouse model, while only smaller NPs (about 100 nm) can cross the BBB in a MCAO‐rat model. Furthermore, NPs without brain targeting ligands were administered i.v. and reduced infarct volume, with improved motor function in rat ischemic stroke models, indicating compromised BBB.

The translation of animal studies to human trials is one of the biggest challenges in the study of neuroprotection in stroke.^[^ ^233^ ^]^ In a personalized stroke therapy, it is important to identify the stage of the stroke. This goal may be achieved by using NPs. These may be employed to identify biomarkers (usually in small quantities) in the brain and help visualization by MRI.^[^ ^233^ ^]^ The following factors should be considered in personalized therapy for stroke: age, infarct size, location, and collateral circulation.^[^ ^233^ ^]^ However, one particular question needs to be addressed: why, after so many NP formulations for stroke, aren't any reaching clinical trial? At the moment a clear answer cannot be provided. However, the intrinsic embolic features of the particles may be an issue, requiring further investigation and resolution to this potential problem. Furthermore, a rapid therapeutic action is required from NPs within few hours from the onset of the ischemia. Finally, the main therapeutic goal is to remove the blood clot in the brain shortly after ischemic stroke.

Hence, the research on NPs perhaps should take these desired effects into consideration.

The above observations indicate that the BBB permeability increases in ischemic stroke, which allows the crossing of small (100 nm) NPs in animal models. However, this is transient.^[^ ^222^ ^]^ Then, perhaps, the secondary compensatory microvascular barrier in ischemic stroke is not as strong as compensatory barriers in MS or AD. However, for effective NC delivery to the brain, these need to be tagged with brain‐targeting ligands as well as ischemic zone‐targeting ligands. It should be noted that the NPs in the systemic circulation may adsorb serum proteins, which is called the formation of protein corona. This protein layer makes NPs susceptible for uptake by macrophages, which would reduce the number of NPs reaching the brain. One way around this could be pre‐coating the surface of NPs with targeting proteins (known as protein corona shield). This approach not only may preserve the targeting ability of the NP, but also may reduce the adsorption of protein serums. This way, the elimination of the NPs may be reduced by macrophages. It should be noted that the targeting ligand itself is a protein with a large mass such as 36.3 kDa.^[^ ^244^ ^]^

### 4.4 Parkinson's Disease (PD)

Lysosomal storage disorders are implicated in pathogenesis of neurodegenerative diseases, notably PD. In this disease, the lysosomes have impaired acidic function and impaired proteolytic enzymes, which lead to dysfunction in clearance and recycling of proteins and cell organelles. Hence, debris start to accumulate in the cells. To alleviate this, NPs provide the opportunity to restore acidic pH and proteolytic activity of lysosomes,^[^ ^245^ ^]^ which form a complex with *α*‐synuclein (*α*‐Sync, a presynaptic neuronal protein that is linked to neuropathology of PD),^[^ ^246^ ^]^ or suppress *α*‐Sync over‐expression through RNA interference.^[^ ^247^ ^]^

Preclinical studies are presented in **Table** **4** for the treatment of PD by using NPs. It can be seen that all routes of administration have been investigated. The NPs had brain‐targeting ligands, when i.v. or i.p. routes were approached. These indicate the relatively high impermeability of the BBB in PD compared to other brain diseases such as the ischemic stroke, although increased permeability of the BBB has been identified in patients with PD.^[^ ^248^ ^]^ Interestingly, PAMAM dendrimers decorated with lactoferrin were able to deliver plasmid DNA (encoding human glial cell‐line derived neurotrophic factor) into following i.v. administration. NPs were effective in improving locomotor activity.^[^ ^249^ ^]^ As long‐term therapy is required for the repair of degenerated neurons and maintenance of the health of existing neurons, then intranasal delivery could provide better patient's compliance compared to i.v. injections. Data in Table 4 shows improved motor activities following intranasal administration, although clinical trial (Phase II) of intranasal reduced glutathione did not show superior improvements in PD motor scores compared to placebo.^[^ ^250^ ^]^

**Table 4:** The summary of NP formulations that have been developed in vitro or in vivo for the treatment of PD

Nanocarrier	Drug	Targeting ligand	Size [nm]	Route [intended route]	Model	Remark	Ref.
PLGA	None	None	50–100	Intracerebral injection	PD mouse model	PLGA‐NP efficiently reduced lysosomal membrane permeabilization in 1‐methyl‐4‐pheynol‐1,2,3,6‐tetrahydropridyine (MPTP)‐injected mice	^[^ ^245^ ^]^
Gold NPs functionalized with chitosan	Plasmid DNA (anti‐*α*‐Syn short hairpin RNA‐encoding)	NGF	10	i.p.	PD mouse model	NPs crossed BBB. NPs significantly recovered the density of the nigra‐striatum compared to untreated PD models	^[^ ^247^ ^]^
PEG‐dendrigraft poly‐l‐lysine (third generation, 123 primary amino groups)	Plasmid DNA (encoding human glial cell‐line derived neurotrophic factor)[hGDNF]	Angiopep‐2 [TFFYGGSRGKR NNFKTEEYC], targets LRP	119 ± 12	i.v.	PD rat models	Animals acquired improved locomotor activity after five injections	^[^ ^252^ ^]^
PEG‐poly(amidoamine) (PAMAM) dendrimer (fifth generation)	Plasmid DNA [hGDNF]	Lactoferrin^a)^	196 ± 10.1	i.v.	PD rat models (6‐hydroxydopamine‐lesioned)	NPs improved locomotor activity of the animals following five alternate day injections (three before the formation of brain lesions and two after brain lesions) NPs significantly enhanced exogenous gene expression in the brain by ≈ 5.2‐folds compared to NPs without lactoferrin	^[^ ^249^ ^]^
PEG‐poly(amidoamine) (PAMAM) dendrimer (fifth generation)	Plasmid DNA [hGDNF]	Lactoferrin	196 ± 10.1	i.v.	PD rat models (rotenone‐induced model)	NPs enhanced expression of hGDNF 4.8 folds in the brain, but less than NPs decorated with lactoferrin	^[^ ^253^ ^]^
PEG‐PLA	Urocortin^b)^	Lactoferrin	120	i.v.	PD rat models	NPs attenuated the striatum lesions and improved behavioral outcomes.	^[^ ^254^ ^]^
Positively charged PLGA	MicroRNA‐124 (surface adsorbed on NPs)	None	210	Intracerebrally	PD mouse model	NPs improved motor activity of the animals	^[^ ^251^ ^]^
Polyethylenimine –dextran sulfate‐Retinoic acid^c)^	Retinoic acid	None	220	Intrastriatal injection	MPTP) induced mouse model of PD	RA‐NPs increased mRNA levels of *Pitx3* (a transcription factor) and resulted in a significant decrease in dopaminergic neuron loss	^[^ ^255^ ^]^
PEG‐PLGA‐odorranalectin^d)^	UCN	None	114.8 ± 5.6	Intranasal	PD rat model	Systemic adsorption of NPS and accumulation in the liver Conjugating NPs with OL increased accumulation of NPs in the brain compared to unconjugated NPs UCN loaded NPs reduced the number of rotations (a behavioral test representing loss of dopaminergic neurons) in rat models of PD compared to untreated control PD model animals	^[^ ^256^ ^]^
Chitosan	Selegilline hydrochloride	Tween 80	303.39 ± 2.01	Intranasal	In vitro	Release of active ingredient over 28 h	^[^ ^257^ ^]^
Gold nanoflowers	l‐DOPA (via LAT‐1)^e)^	l‐DOPA	90	i.v.	In vitro	The NPs did not induce neuroinflammation.	^[^ ^258^, ^259^ ^]^

^a)^ Mammalian cationic iron‐binding glycoprotein, which belongs to the Tf family

^b)^ Urocortin (UCN), a corticotropin‐releasing hormone family of peptides (4 kDa, around 40 amino acids) with restoration of nigrostriatal function property

^c)^ Retinoic acid (RA) plays an essential role in the commitment, maturation and survival of neural cells

^d)^ Odorranalectin (OL) is a small molecule of the lectin family with minimal immunogenicity and bioadhesive properties

^e)^ LAT‐1: Large neutral amino acid transporter.

To enhance neurogenesis in the subventricular zone of the adult mammalian brain, Saraiva et al. formulated PLGA NPs with positively charged surfaces using protamine sulfate. MicroRNA‐124 was surface adsorbed to these NPs and administered intracerebrally into mouse models of PD, with outcomes showing miRNA‐124 NPs improved motor activity of the animals.^[^ ^251^ ^]^

Recombinant human platelet‐derived growth factor‐BB (rhPDGF‐BB) reduced PD symptoms and increases dopamine transporter binding in animal models.^[^ ^260^ ^]^ In a phase I clinical trial rhPDGF‐BB was injected intracerebroventricularly into 12 PD patients at the maximum dose of 5 µg per day for 12 days. At the end of the trial, all patients had improved motor examination scores.^[^ ^261^ ^]^ In terms of using NPs in the clinic for the treatment of PD, CERE‐120 is an experimental AAV that was engineered to deliver the human gene for neurturin. This vector was also delivered via intracerebroventricular injection.^[^ ^262^, ^263^ ^]^ CERE‐120 was investigated in two clinical trials with 50 patients. The outcomes were not better than placebo; however, there were improved measures that indicated potential benefits. Therefore, CERE‐120 was investigated in another trial (Phase II) with ≈52 patients (https://clinicaltrials.gov/ct2/show/NCT00985517). In addition gold nanocrystals (CNM‐Au8) have been developed with the size of 13 nm^[^ ^264^ ^]^ that are administered orally to patients with Parkinson's disease. CNM‐Au8 NPs are clean‐surfaced, faceted nanocrystals of gold, which upon administration distribute in organs including the brain and promote the oxidation of nicotinamide adenine dinucleotide hydride (NADH) to the critical energetic co‐factor, NAD^+^. NADH oxidation drives cellular respiratory and metabolic processes that play key roles in the brain energetically demanding process of myelination.^[^ ^264^ ^]^ Therefore, these gold nanocrystals will change the brain metabolism to repair damages. CNM‐Au8 nanocrystals are now in Phase 2 clinical trial (NCT03815916).

The above studies do not indicate significant breakdown of the BBB in PD. Hence, nasal delivery, use of brain targeting ligands, or intracerebral injections was employed to cross/bypass the BBB for the delivery of NPs in PD. Therefore, NPs should have brain targeting ligands as well as suitable dimensions (preferably less than 120 nm, but ideally 10 nm) to cross the BBB and reach the brain parenchyma via i.v. injections or oral administration. The choice of brain targeting ligand is important, as lactoferrin may allow larger NPs (about 200 nm) to cross the BBB compared to transferrin conjugated NPs.^[^ ^265^ ^]^

### 4.5 Brain Tumors

Brain tumors are categorized to primary and secondary intracranial tumors.^[^ ^266^ ^]^ Primary tumors arise from cells within the CNS,^[^ ^267^ ^]^ while secondary tumors are metastases from cancers elsewhere in the body.^[^ ^266^ ^]^ Primary brain tumors account for 1.6% of all cancer cases,^[^ ^268^ ^]^ and malignant gliomas are the most deadly and common brain tumors.^[^ ^269^ ^]^ Gliomas arise from glial or precursor cells and include astrocytoma (including glioblastoma), oligodendroglioma, ependymoma, oligoastrocytoma (mixed glioma), and malignant glioma.^[^ ^270^ ^]^ Among gliomas, glioblastoma accounts for the majority of gliomas^[^ ^270^ ^]^ and is the most lethal primary brain tumors in adults,^[^ ^267^ ^]^ with a median survival of 16 months.^[^ ^271^ ^]^ As a result, intensive research has been conducted to identify suitable treatments for glioblastoma. In the following section, part of recent NP based therapeutic formulations is discussed. It should be noted that the BBB is relatively intact at the early stages of developing brain tumors.^[^ ^272^ ^]^

Carbon nanotubes (CNTs) have cylindrical shape and are composed of graphene sheets with sp^2^ hybridized carbon atoms. Among drug delivery systems, CNTs have emerged as promising platforms to cross the BBB and deliver therapeutic agents to brain tumors. CNTs can have single (SWCNT) or multiple walls (MWCNT).^[^ ^273^ ^]^ Malignant gliomas are the most common fatal brain tumors, due to their ability to escape a local immunosuppressive microenvironment. To overcome this, CpG oligodeoxynucleotides (Toll like receptor 9 agonists) were conjugated with SWCNTs, and delivered by intracranial injection to mice bearing intracranial GL261 gliomas. Animals remained tumor‐free for more than three months.^[^ ^274^ ^]^ Fan et al. also employed CpG conjugated CNTs for the treatment of metastatic brain tumors in mice bearing brain B16.F10 melanomas as well as subcutaneous tumors. The NPs were administered by intra‐tumoral injections by stereotactic administration. It was found that mice recovered from both tumors, showing that an improved immunosuppressive response in the brain microenvironment was sufficient to suppress the subcutaneous tumor too.^[^ ^275^ ^]^ Chemically functionalized MWCNT were able to cross the BBB at significant amounts between 1 and 4 h post i.v. injections. The NPs had diameter of 9.2 ± 2.7 nm with a length of 396 ± 290 nm. The NPs were not conjugated with brain targeting ligands.^[^ ^276^ ^]^

**Figure 9:**
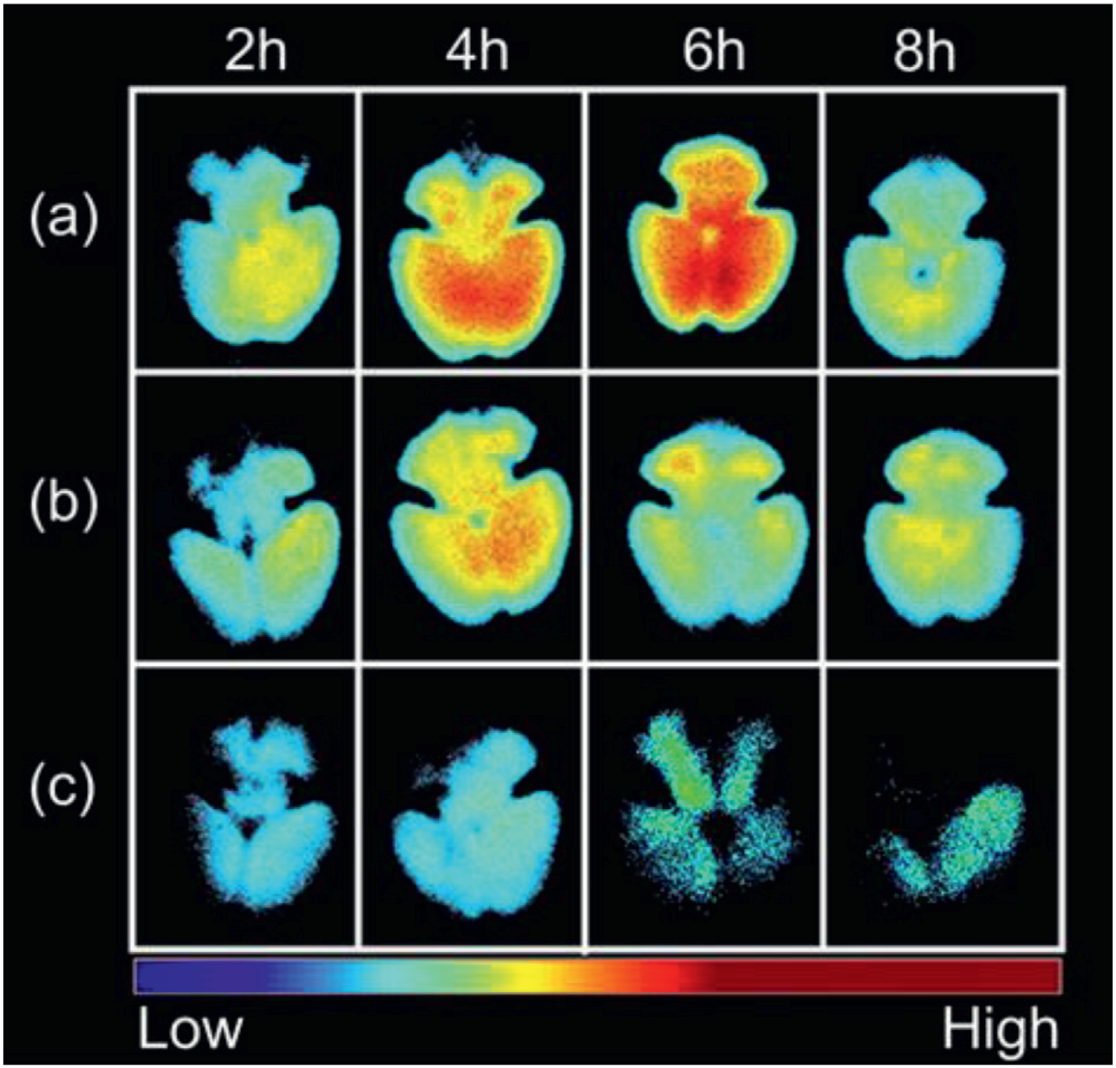
In vivo fluorescence imaging of brains collected from Balb/c mice after intravenous injection at a dose of 5 mg DOX‐equiv. kg^−1^ body weight. a) DOX‐O‐MWNTs‐PEG‐ANG group is shown on the upper row, b) DOX‐O‐MWNTs‐PEG group is on the middle row, and c) DOX group is on the lower row. It can be seen that NCs without targeting ligands achieved a considerable accumulation in the brain. Reproduced with permission.^[^ ^277^ ^]^ Copyright 2012, Elsevier.

Oxidized multi‐walled carbon nanotubes were conjugated with angiopep‐2 (TFFYGGSRGKRNNFKTEEY) by using DSPE‐PEG2000‐MAL and loaded with doxorubicin (DOX‐O‐MWCNT‐PEG‐ANG). Angiopep‐2 targets the LRP receptor on the BBB as well as glioma cells. The NCs had 10 nm diameter with the length of 120 nm. DOX‐O‐MWCNT‐PEG‐ANG NCs were administered i.v. to mice bearing intracranial C6 glioma. The median survival time for DOX‐O‐MWCNT‐PEG‐ANG NCs was 43 days, better than the 36 days for angiopep‐free DOX‐O‐MWCNT NCs. In vivo fluorescence imaging of brains also showed slight decrease of fluorescence intensity for DOX‐O‐MWCNT NCs compared to DOX‐O‐MWCNT‐PEG‐ANG formulation (**Figure** **9**).^[^ ^277^ ^]^ This study also indicates that NPs around 100 nm may target brain parenchyma through compromised BBB and EPR effects brought about by the glioma. Figure 9 shows dispersion of DOX‐O‐MWCNT NCs in other regions of the brain as well as the tumor, suggesting redistribution of the NPs in the brain due to the CSF recirculation in the brain.

However, neurotoxicity is the main obstacle in the application of CNTs for the treatment of brain diseases.^[^ ^278^, ^279^, ^280^ ^]^ CNTs affect brain neurons: CNTs were found to have negative behavioral effects including increased pacing distances and times necessary to locate a platform following i.p. injection into rats.^[^ ^281^ ^]^ It has also been shown that functionalized CNTs caused the release of inflammatory cytokines in the mouse brain, following cortical stereotactic administration.^[^ ^282^ ^]^ These observations may be the reason that there have been no clinical trials for the use of CNTs for the treatment of brain glioblastoma.

Liposomes containing doxorubicin have also been employed in the treatment of brain tumors in mice. The surface of liposomes was decorated with a tumor‐specific pH‐responsive peptide H_7_K(R_2_)_2_ (Ac‐RRK(HHHHHHH)RR‐NH2), and the antitumor activity of the liposomes (DOX‐PSL‐ H_7_K(R_2_)_2_) were investigated in C6 tumor‐bearing nude mice. The liposomes had a size of 92 ± 3.9 nm.^[^ ^283^ ^]^ These were administered i.v. and reduced significantly the growth of tumors. In addition, liposomes without H_7_K(R_2_)_2_ targeting peptide reduced significantly the growth of tumors compared to control animals, but not as much as DOX‐PSL‐ H7K(R_2_)_2_ liposomes.^[^ ^283^ ^]^ It should be noted that the peptide is specific to tumors not the BBB.

In another study, enzyme responsive NPs were developed for the treatment of glioma. These NPs had a core made of gelatine, degradable by MMP‐2. The surface of the gelatine NPs was conjugated with gold NPs carrying doxorubicin and a glioma‐targeting peptide RRGD (G‐AuNPs‐DC‐RRGD), where RRGD presents the conjugation of R8 (RRRRRRRR) to cyclic RGD peptide with a terminal cysteine (Cys‐c(RGDfK), cysteine was conjugated to the branch of lysine).^[^ ^284^ ^]^ The NPs originally had a size of 188 nm, but in the presence of MMP‐2, they shrank to 55 nm. I.v. injection of G‐AuNPs‐DC‐RRGD NPs led to the accumulation of the NPs in the glioma of C6 cell xenograft‐bearing mice. The NPs started to accumulate in the tumor 4 h post injection.^[^ ^285^ ^]^ These observations may suggest that parts of NPs were digested by serum MMP‐2 enzyme and the accumulation of NPs in the glioma was due to their subsequent reduced size. In this regard, enzyme responsive NPs have been developed that contained monoclonal antibodies for the treatment of brain tumors.^[^ ^286^ ^]^ These NPs were decorated with 2‐methacryloyloxyethyl phosphorylcholine targeting two receptors on the BBB: 1) the nicotinic acetylcholine receptor, 2) the choline transporter. The core of NPs contained a peptide (VPLGVRTK) responsive to tumor‐derived MMP‐2. The NPs had an average diameter of 25 nm, and these crossed the BBB following i.v. injection and degraded in the brain by tumor‐derived MMP‐2. The administration of NCs containing mAbs resulted in significantly higher CSF mAb concentrations in comparison to those receiving mAbs alone. For example, mice injected with native nimotuzumab showed a CSF concentration of 0.084 µg mL^−1^ at 12 h which corresponded to 0.1% of the plasma concentration. Conversely, the CSF concentration of the mice injected with mAb NCs was tenfold higher at 12 h (0.85 µg mL^−1^) than those treated with native nimotuzumab. This corresponded to 1.1% of the plasma concentration.^[^ ^286^ ^]^ These observations suggest that both size and the presence of brain targeting ligands (for two receptors) on the NPs played key roles in high brain delivery of mAbs. It should be noted that the molecular weight of MMP‐2 (72 kDa)^[^ ^287^ ^]^ is close to bovine serum albumin (66.5 kDa, hydrodynamic diameter: 6.8 nm) or hemoglobin (64.5 kDa, hydrodynamic diameter: 5.5 nm),^[^ ^288^ ^]^ hence, the hydrodynamic diameter of MMP‐2 would be around 7 nm. Therefore, enzyme‐responsive NPs should have channels greater than 7 nm to allow the access of this enzyme to the cleavable sites.

Legumain is a lysosomal cysteine protease,^[^ ^289^ ^]^ which is localized in lysosomes and endosomes.^[^ ^290^ ^]^ It is suggested that legumain extracellular levels increase in cancer.^[^ ^290^ ^]^ Based on this gold NPs have been developed that are responsive to legumain. In this approach gold NPs are linked via a peptide sequence (Ala‐Ala‐Asn‐Cys‐Lys) responsive to legumain. These gold NPs were administered with other gold NPs, which carried 2‐cyano‐6‐amino benzothiazole on the surface (AuNPs‐CABT). Following i.v. injection of these NPs (no brain‐targeting ligand) to orthotopic C6 glioma‐bearing mice, the NPs were accumulated in the brain tumor through the EPR. Legumain cleaved the Ala‐Ala‐Asn‐Cys‐Lys peptide and exposed 1,2‐thiolamino groups on cysteine, which reacted with the cyano groups 2‐cyano‐6‐amino benzothiazole on the surface of AuNPs‐CABT NPs via click cycloaddition. This conjugation led to the aggregation of gold NPs and, therefore, prevented the return of NPs back to the blood. In one formulation, gold NPs carried doxorubicin on the surface, which resulted in improved survival of the animals.^[^ ^291^ ^]^ It should be noted that the accumulation of gold NPs in the body may have negative effects on the gold NP accumulation organs such as the liver.^[^ ^292^ ^]^

There are NP‐based drug delivery systems for the treatment of brain tumors that have made their ways to clinical trials (**Table** **5**). For example, AGuIX NPs have been in clinical trials (registrations: NCT04094077, NCT02820454, NCT03818386). These NPs have sub‐5 nm sizes and are made of a polysiloxane matrix and gadolinium chelates. These NPs are injected intravenously, and cause radiosensitization of the cells that uptake these NPs. Radiotherapy of these cells cause the release of ROS from lysosomes, which leads to cell apoptosis.^[^ ^293^ ^]^ One of the main advantages of AGuIX NPs is rapid clearance from the body via kidneys and lack of accumulation in the liver.^[^ ^294^ ^]^ Interestingly, many of NP formulations do not have brain targeting ligands (Table 5). Then brain drug delivery would be via the EPR. Only folic acid and antibodies have been employed as brain targeting ligands. The lack of safety data for peptide‐based brain targeting ligands could be one of the reasons for them not being in clinical trials. Most of the NP formulations have sizes less than 200 nm.

**Table 5:** Clinical studies on nanoparticles in brain cancer

NP	Drug	Brain targeting ligand	Size [nm]	Route	Phase	Indication	Ref./study number
Gold	Spherical nucleic acid (NU‐0129)	None	175	i.v.	Early Phase 1	Recurrent glioblastoma targeting BCL2L12	^[^ ^298^ ^]^ NCT03020017
Albumin	Rapamycin	None	100	i.v.	Phase 2	High grade recurrent glioma and newly diagnosed glioblastoma	NCT03463265
Nanoliposomes	CPT‐11	None	9–101	i.v.	Phase 1	Glioblastoma	^[^ ^299^ ^]^ NCT00734682
EGFR(V)‐EDV	Doxorubicin	Bispecific targeted antibodies to leaky blood vessels of tumor	400	i.v.	Phase 1	Glioblastoma	^[^ ^300^ ^]^ NCT02766699
Liposome	p53 cDNA	Folic acid	<100	i.v.	Phase 2	Glioblastoma	^[^ ^301^, ^302^ ^]^ NCT02340156*
Polysiloxane Gd‐Chelates	AGuIX	None	3.4–5.5	i.v.	Phase 1	Brain metastases	^[^ ^293^ ^]^ NCT02820454
Polysiloxane Gd‐chelates	AGuIX	None	5	i.v.	Phase 2	Brain metastases	NCT04094077
Polysiloxane Gd‐chelates	AGuIX	None	5	i.v.	Phase 2	Brain metastases, adult	NCT03818386
Silica	124I‐cRGDY‐PEG‐dots	None	6–7	i.v.	Microdosing study	Malignant Brain tumors	^[^ ^303^ ^]^ NCT01266096
Iron oxide	Ferumoxytol	None	17–31	i.v.	Phase 1	Brain neoplasms	^[^ ^304^ ^]^ NCT00769093
Albumin bond paclitaxel (ABI‐007)	Paclitaxel	None	130	i.v.	Phase 1	Brain and central nervous tumors	^[^ ^305^ ^]^ NCT00313599
Nab paclitaxel	Paclitaxel Combinational therapy with 9‐ING‐41 (potent GSK‐3*β*)	None	130	i.v.	Phase 1/2	Refractory brain tumors, glioblastoma multiform, malignant glioma	NCT03678883
Gold	Panobinostat	None	26^a)^, ^b)^	Convection‐enhanced delivery (CED)	Phase 1	Diffuse midline gliomas	^[^ ^306^ ^]^ NCT04264143
EDV‐nanocells	Doxorubicin	Bispecific antibodies for tumors	400 ± 20	i.v.	Phase 1	Glioblastoma	^[^ ^307^ ^]^ NCT02766699

^a)^ The size is based on the formulation of P407 micelles

^b)^ Based on other trials, the actual registration does not specify.

In a recent work, real time‐MRI was employed to guide a catheter via intra‐arterial administration to the proximity of the brain tumor. Then the BBB was opened locally by administration of mannitol, which was followed by administration of bevacizumab to be taken up by the tumor.^[^ ^295^ ^]^ This approach may be applied for drug delivery to the brain by NPs.

The above studies suggest the increased permeability of the BBB in brain tumors. This can be supported by a recent work that major facilitator domain containing protein 2A (Mfds2a) is enriched in CNS vasculature, and Msfd2a helps BBB maintain impermeability by reducing the EC transcytosis transport mechanism.^[^ ^296^ ^]^ Loss of Mfsd2a in brain endothelial tumors resulted in increased BBB leakage.^[^ ^297^ ^]^ The above studies indicate that NCs in sizes less than 100 nm would cross the BBB in the malignant regions without needing to employ brain‐targeting ligands, although the presence of these ligands improves drug delivery to the brain.

### 4.6 BBB Leakage in Other Diseases

As well as the above brain diseases, the BBB permeability increases in other diseases too. In the following part of these are explained. Vascular bags were observed around the ECs of patients with cerebral small vessel disease, driving remodeling of microvasculature.^[^ ^308^ ^]^ In the brain tissues of autism spectrum disorder (ASD) subjects, levels of MMP‐9 are significantly increased^[^ ^309^ ^]^ contributing to an impaired BBB. In diabetes, there are changes to the microvascular structure of the brain, and it has been shown in a mouse model of diabetes that the BBB changes by a reduction in the number of astrocytes and degeneration of pericytes.^[^ ^310^, ^311^ ^]^ In vitro studies showed that inflammation (acute bacterial infection) caused the BBB to become permeable to albumin,^[^ ^312^ ^]^ however, in vivo studies showed that this was not always the case.^[^ ^313^ ^]^

It needs to be mentioned that the BBB presents different receptor profiles, depending on the disease. In this regard, Chen et al. demonstrated that brain targeting ligands should be chosen based on the brain disease since a particular brain targeting ligand may be suitable for one disease but not for another. This study found that AAVs presenting a WPFYGTP epitope showed 35 times more efficiency in targeting the brain than the liver. Interestingly, AAVs with epitopes for brain microvessels of wild‐type mice were not effective for the mucopolysaccharidosis type VII mouse brain. This study also shows that brain neurons and parenchyma may be supplied with enzymes by targeting brain microvessels with AAVs carrying genes for those enzymes.^[^ ^314^ ^]^

Hypertensive encephalopathy^[^ ^315^ ^]^ and acquired immune deficiency syndrome (AIDS) result in BBB disruption.^[^ ^316^, ^317^ ^]^ In diseases such as AIDS, where movement of immune cells (in and out of the CNS) is enhanced, BBB disruption is evident due to diapedesis (the passage of blood cells through the intact walls of the capillaries).^[^ ^318^ ^]^ Chronic pain and intense inflammation in animal models have been reported to cause BBB disruption.

It has been suggested that BBB permeability increases in ornithine transcarbamylase deficiency,^[^ ^319^ ^]^ with excess ammonia crossing the BBB in these patients and being converted into glutamine. The excess glutamine increases brain osmotic pressure, which leads to brain edema and neurological damage.^[^ ^320^ ^]^ To alleviate the excess ammonia in circulating blood, PEGylated liposomes (MRT5201) have been developed with the size of 16 nm that encapsulate codon‐optimized human ornithine transcarbamylase mRNA to target the liver.^[^ ^321^, ^322^ ^]^ These NPs are now in a clinical trial (NCT03767270), although the clinical trial is on hold for further toxicology data on clearance time.^[^ ^323^ ^]^

In conclusion, the BBB disruption occurs in other diseases apart from the main neurodegenerative diseases. Therefore, NP based drug delivery systems may be developed to minimize neurological damages due to increased BBB permeability. Most importantly, the BBB receptor profile changes with the disease, and this may require optimizing the brain targeting ligands based on the modified version of BBB receptor profile.

## 5 In Vitro Methods for the Assessment of Drug Delivery to the Brain

Permeability of brain microvasculature contributes significantly to the efficacy of drugs at sites within the CNS. Therefore, there is much effort to understand the BBB, to simulate its barrier mechanisms and to model therapeutic delivery strategies, pathologies, or toxicological insults that may overcome them. The preclinical development of drugs directed toward CNS targets for the treatment of CNS diseases involves complementary modeling methods. These include in vivo (live animal), in vitro (cell culture) and in silico (computation based on physiochemical and physiological parameters) approaches.

Although in vivo animal models are considered the gold standard of preclinical predictive tools,^[^ ^324^ ^]^ it is estimated that 80% of drug candidates identified this way later fail in clinical trials.^[^ ^325^ ^]^ In vivo work is relatively low throughput, expensive and limited by ethical considerations. In vitro models that attempt to recapitulate the anatomical and physiological barrier properties, which determine drug delivery across the BBB, are therefore desirable to inform in vivo work and justify clinical developments. However, no standard in vitro screen has yet been agreed for assessing brain uptake,^[^ ^324^, ^326^ ^]^ rather modifications and different methods are continually being proposed to improve simulations of barrier characteristics.^[^ ^327^ ^]^

**Figure 10:**
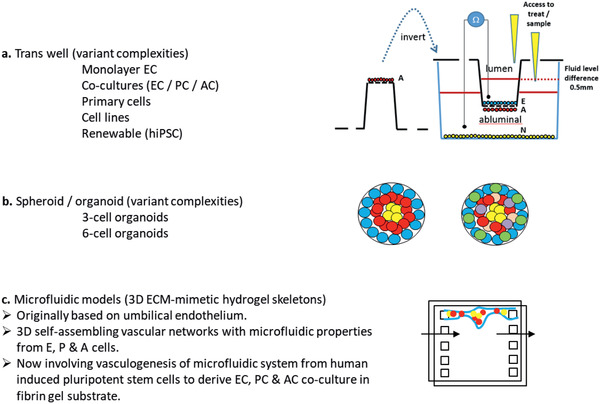
Outlines of three types of in vitro BBB models currently available.

Here we review, using illustrative examples, developments in alternative in vitro methods to simulate the BBB and experimental designs that have validated them as tools to assess drug delivery. A binary terminological distinction of “Static” versus “Dynamic” methods is commonly used, based on the understanding that barrier phenotypes are induced and maintained by, not only, a) the cellular contacts made by EC with other cells of the NVU, notably pericytes/vascular smooth muscle cells and astrocytes, but also, b) the shear forces imparted by circulating plasma on ECs. Three main categories of in vitro model are encountered (**Figure** **10**), as follows:

The two main “Static” models (without fluid flow) comprise: a) Transwell—a suspended semipermeable platform on which an endothelial monolayer is grown and b) Spheroid—a multicellular organoid of brain cells enveloped in endothelia, while c) “Dynamic in vitro (DIV) models” include those of sub‐millimeter lumen diameter, but more recently their smaller iteration, hence the term microfluidic devices. These comprise synthetic scaffolds enabling organotypic growth of cells into perfusable microvasculature networks, also termed “organ‐on‐a‐chip.”^[^ ^328^ ^]^

### 5.1 Transwell Models of the BBB

Transwell methods have become the most commonly used and convenient in vitro approach.^[^ ^329^, ^330^, ^331^, ^332^, ^333^ ^]^ They involve the suspension of a permeable platform as an insert into wells of multiwell plates. ECs are grown on the matrix‐coated (collagen/fibronectin) surface of the insert to form a monolayer that is suspended above the base plastic of the well, giving access to two compartments: luminal, drug delivery site (inside the insert) and abluminal (beneath the insert) equivalent to the tissue parenchyma.

#### 5.1.1 Monoculture of Endothelium on Transwell Inserts

For convenience, ease of tissue sourcing and robust barrier properties, porcine or bovine brain EC (PBEC) monolayers are an impressive option.^[^ ^334^ ^]^ When grown in monolayers, primary PBEC can achieve trans‐endothelial electrical resistance (TEER) of 800 Ωcm^2^ within 24 h, a functional measure of paracellular junctional tightness.^[^ ^334^ ^]^ They can also be cocultured with astrocytes to enhance expression of other barrier features, such as receptor‐mediated transcytosis.

There is support for the validity of transwell animal models of the BBB. Heymans et al. have compared the in vitro and in vivo permeability of a panel of 27 CNS drugs having a wide range of physiochemical properties and classified by properties such as: a) their binding to brain tissue (fraction unbound to brain, *f*_u,br_, the ratio of drug in interstitial fluid/total in brain) and b) their lipophilicity (LogP).^[^ ^335^ ^]^ These authors used two alternative in vitro bovine BBB models^[^ ^336^ ^]^ and correlated drug permeabilities in these with published data from an in vitro method of rat in situ brain perfusion, employing the same drugs.^[^ ^337^ ^]^

The two alternative in vitro transwell BBB models used primary bovine BCECs grown on transwell semi‐permeable inserts as a monolayer, either a) in monoculture, else b) in quadruple coculture with mixed primary glial cells grown in the well to which the monolayer insert was placed. Glia were mixed in the following proportions of cells that could be identified/followed by cell‐specific immune‐fluorescent markers: 60% glial fibrillary acidic protein positive astrocytes, 30% O4 positive oligodendrocytes and 10% ED‐1 positive microglia. The latter better modeled not only permeability across the BBB but also the binding kinetics of drug to brain tissue.^[^ ^335^ ^]^

Alternative calculations of in vitro brain permeability were made: 1) Apparent permeability coefficient (*P*_app_) which was defined as the rate of flux/appearance of compounds in the abluminal well (receiver) compartment 30 min following initial delivery into the luminal insert (donor) compartment. 2) Endothelial permeability (Pe) was calculated by the method of cylindrical pore theory, involving average cumulative clearance of drug over time to determine the permeability surface area product of the filter alone and that of the filter with endothelia. The difference being divided by surface area of the filter insert to give Pe.^[^ ^335^ ^]^

Good correlation between in vitro and in vivo drug permeability was reported,^[^ ^335^ ^]^ suggesting the in vitro BBB model to be predictive of in vivo rate of brain penetration for those CNS compounds used; but that the correlation was best when the Pe calculation of in vitro permeability was used. Also, modeling discrepancies for drugs having a low fraction unbound to brain (*f*_u,br_) were resolved when using the coculture method, consistent with the hypothesis that the presence of glial cells in the abluminal compartment better mimicked the effect of brain tissue binding that occurs in vivo.

#### 5.1.2 Multiculture of Neurovascular Cells in Transwell, Using Primary, Immortalized, or “Renewable” Stem (Isolated or Induced) Human Cells

Though regarded as a valuable species comparison with human physiology, the favorable barrier properties that can be achieved when using bovine or porcine endothelia in transwell are not readily duplicated using human cells without more complexity of coculture conditions. Human tissues are also not as readily available as animal. Nevertheless, renewable human cells include hematopoietic stem cells from umbilical cord blood,^[^ ^338^ ^]^ circulating endothelial progenitors mobilized from bone marrow^[^ ^339^ ^]^ and induced pluripotent stem cells^[^ ^340^, ^341^ ^]^ (hiPS, e.g., the lines IMR90‐4 and ARiPS). These can be differentiated into somatic cell types, thereby offering a virtually unlimited cell source with which to model BBB in vitro.

Appelt‐Menzel et al. have compared the effects of the combination of different cell types in bi‐, tri‐ and quadruple coculture in transwell on the barrier properties of human EC derived from hiPS.^[^ ^342^ ^]^ They obtained five human cell types: i) primary fetal brain pericytes, ii) fetal NSC as well as induced pluripotent stem cells (hiPS) induced to differentiate into iii) NSC, iv) ECs, and v) astrocytes using methods derived from previous works.^[^ ^343^, ^344^ ^]^ Treatment of EC grown in monolayer on transwell inserts with 10 × 10^−6^ m RA was used to enhance their barrier properties.^[^ ^345^ ^]^

Transwell coculture is limited in that it simulates “indirect, humoral, noncontact” effects of associated brain cell types on BBB EC integrity. Transwell cocultures may simulate only the humoral interactions between cells associated with the BBB, even if cocultured cells are plated on the abluminal surface, below ECs on the luminal surface of the insert. Nevertheless, Appelt‐Menzel et al. investigated the beneficial effects of combination of ECs in bi‐, tri‐, and quadruple cocultures with pericytes, astrocytes and NSCs in transwell. hiPS‐derived ECs were grown on the luminal transwell inserts while all other cells were plated, not in direct contact with ECs, but within the well base, i.e., not upon the abluminal surface of the transwell. This facilitated uptake assays, since absorbance by cell types other than ECs could be avoided.^[^ ^342^ ^]^ Although this format does not fully replicate the direct cellular contacts found in 3D spheroid organoids or microfluidic organoids (subsequent sections) it has experimental benefits.

The barrier properties of hiPS‐derived ECs obtained were validated by their expression of EC markers such as von Willebrand factor (vWF), TJ associated protein (ZO‐1), and the GLUT‐1; also the adherence junction protein, vascular endothelial cadherin (CDH5); though there was weaker expression of the endothelial proteins angiopoietin receptor 2 (TIE2) and PECAM1 (CD31).^[^ ^342^ ^]^ EC also expressed BBB transporters and TJ proteins (the P‐gp efflux transporter, *ABCB1* (efflux transporter), the *SLC1A1* (glutamate transporter), *SLC2A1* (glucose transporter), and *OCLN* (occludin)). An uptake assay with fluorescein isothiocyanate FITC‐labeled acetylated low‐density lipoprotein also indicated lower accumulation compared to human umbilical vein endothelial cell monolayer as control.^[^ ^342^ ^]^

Electrical resistance has been measured up to 5900 Ωcm^2^ in vivo, across the BBB in anaesthetized rats.^[^ ^346^ ^]^ The TEER of triple cocultures of hCMEC/D3 in transwell has been reported as only ≈50 Ωcm^2^.^[^ ^347^ ^]^ This is comparable to other studies of immortalized human ECs in transwell, suggesting that the electrical resistance of human endothelia is low compared to other species, notably including murine, bovine and porcine. However, in the study by Appelt‐Menzel et al. using human cells, the mean TEER for the EC monolayer itself, and all nine cocultures in bi, tri, and quadruple combinations of human cells in vitro, were greater than 1000 Ωcm^2^, indicating tightness of cellular junctions limiting paracellular permeability that can be achieved in transwell systems.^[^ ^342^ ^]^

The effect of coculture was to significantly increase TEER compared with hiPS‐EC monolayers (1198 ± 265 Ω cm^2^) for both tri‐culture of EC with hiPS‐NSC and primary human pericytes (1723 ± 90 Ω cm^2^) or quadruple coculture with hiPS‐NSC, astrocytes and primary human pericytes (1757 ± 320 Ω cm^2^).^[^ ^342^ ^]^ The expressions of transporters (ABCB1, SLC1A1, SLC2A1) and the TJ component occludin were on average upregulated by 1.5‐fold following quadruple culture, but statistical significance was reached only for SLC2A1.^[^ ^342^ ^]^

Investigation of drug transport via transcellular routes revealed that compared to EC in monocultures, quadruple cultures significantly decreased permeability to caffeine, a marker of fast transcellular trafficking.^[^ ^342^ ^]^

Appelt‐Menzel et al. illustrated the utility of transwell endothelial monolayers for in vitro simulation of human BBB. EC barrier properties were augmented by retinoic acid (RA)‐dependent differentiation from hiPCs, and while kept from making direct contacts with EC, indirect mediation from coculture with tri‐ and quadruple combinations of pericytes or astrocytes and NSCs promoted barrier expression.^[^ ^342^ ^]^ Although the transwell method does not model the possible effects of fluidic phenomena and direct cellular contacts, it provides a robust validation that renewable human in vitro BBB modeling can be achieved by this “static” in vitro system. Moreover, the utility of the transwell method is suggested by its popularity.

### 5.2 Spheroids—Multicellular Neurovascular Spherical Organoids as In Vitro BBB Models

To validate a relatively new in vitro system, Cho et al. have compared the barrier characteristics of triple coculture of the same cells in transwell with self‐assembling multicellular organoids (spheroids).^[^ ^347^ ^]^ To form spheroids the following cell types were combined in triple coculture on low attachment plates, coated with soft agarose, in 1:1:1 ratio,^[^ ^347^, ^348^ ^]^ but with the omission of VEGF‐A supplement, known to reduce paracellular barrier integrity:

- Primary human astrocytes (phAC)
- Primary human brain vascular pericytes (pHBVP) and
- Either a) primary human brain microvascular EC (phBMVEC) or b) an immortalized human cerebral microvascular EC line D3 (hCMEC/D3).

Cells formed compact spheroids within 12 h, with average success rate of 90% based on physical characteristics. Spheroids remained stable and viable over 17 days. After 48 h coculture, stratification of cells were made using fluorescence dyes (cell tracker dyes). After 48 h coculture, stratification of cells were made using fluorescence dyes (cell tracker dyes). The cells were fluorescently labeled for long‐term tracing of living cells. The spheroids were imaged in confocal laser scanning microscopy.^[^ ^348^ ^]^ Astrocytes occupied the spheroid core while ECs together with pericytes formed a surface monolayer, mimicking the in vivo organization of these cells. Consistent with the anatomical formation of a paracellular barrier, expression of TJ proteins (occludin, claudin 5 and ZO‐1) was observed at the surface of spheroids.

In terms of functional properties, the surfaces of spheroids established with either primary or immortalized ECs exhibited low permeability to high molecular weight TRITC‐dextran (155 kDa).^[^ ^347^ ^]^ The paracellular permeability of spheroids to TRITC‐dextran (10 mg mL^−1^) was responsive to the agonist VEGF‐A, which was shown to affect paracellular permeability by disrupting ZO‐1 staining. Exclusion transport appeared intact/active in spheroids, since treatment with an inhibitor of P‐gp efflux (LY335979)^[^ ^347^ ^]^ significantly increased the influx of the fluorescent substrate rhodamine 123 (Rho 123) into spheroids. In this work, active transport of fluorescently labeled (Cy5.5 or TRITC) brain penetrant agent angiopep‐2 via RMT through the LRP‐1 was demonstrated by optical sectioning of spheroids in confocal microscopy. Penetration to 100 µm was significantly greater for angiopep‐2 than for a scrambled sequence of amino acids in spheroids prepared from either hBMVECs or hCMEC/D3 cells. That spheroids remained completely impermeant to TRITC‐dextran (155 kDa; 10 µg mL^−1^) suggested receptor mediated transport rather than nonspecific permeabilization. Further, angiopep‐2 accumulation by spheroids was inhabitable when incubated at 4 °C. Fluorescence microscopy of frozen section confirmed angiopep‐2 in the spheroid core. Delivery of angiopep‐2 conjugates of various sizes (6, 8, and 30 kDa) to the spheroid core was observed using tetramethylrhodamine (TAMRA) labeling. This spheroid model therefore demonstrates the potential of angiopep‐2 to act as a vehicle to facilitate drug permeation across the BBB.

Since most drugs are not fluorescent, their transit is difficult to follow. Unlike the aqueous superfusing compartments of transwells that are conveniently sampled, making them amenable to chemical analysis, including high performance chromatography,^[^ ^349^ ^]^ to verify drug transit, the monitoring of drug movements into spheroids is more difficult. A novel method, matrix‐assisted laser desorption/ionization mass spectrometry imaging (MALDI‐MSI) has therefore been applied at a spatial resolution of 30 µm in frozen section of spheroids (avg. diameter ≈300 µm) to confirm the penetration of BKM120 (a BBB‐penetrant control)^[^ ^347^, ^350^ ^]^ versus no detectable signal of dabrafenib (BBB‐impenetrant control). Penetration occurred in the presence of no increase in TRITC, confirming paracellular integrity.

#### 5.2.1 Validation of Spheroids as Models of Pathology and Toxicological Insult

In an effort to recapitulate the full range and proportionate complement of cells found in vivo, Nzou et al. combined the six major types present within human brain cortex in an in vitro spheroid model of the BBB. Organoids (spheroids) were prepared by the hanging drop method (rather than low adherence substrate) relying on the self‐assembly of cells. They determined that combining cells en masse (altogether) was less effective at creating a contiguous endothelial surface coat than a two‐stage process; first growing a central core of astrocytes and neurones prior to coating with endothelia/pericytes.^[^ ^351^ ^]^ The cells used were either 1) primary human brain microvascular endothelial cells (HBMEC); 2) pHBVP, and the following derived from hiPSC: 3) human astrocytes (HA); 4) human microglia (HM); 5) human oligodendrocytes (HO), and 6) human neurones (HN). Cells were expanded before being harvested and then combined as explained in the following.

In preliminary experiments, 3‐cell (HBMEC, HP, HA) and 4‐cell organoids were made using primary HBMEC, HP, HA, and HCN‐2 (the ATCC cell line: homosapiens cortical neurones‐2) prelabeled with a long‐term tracker dye, in the ratio of 1:1:5:6. These were allowed to mature for 96 h before being placed individually into 96 well plates for use. That incomplete barrier properties were obtained was demonstrated by FITC‐labeled IgG being visualized at the spheroid core, though labeling was less than following paracellular permeabilization by histamine treatment.^[^ ^351^ ^]^

In order to prepare 6‐cell organoids a staged method was used. This overcame incomplete EC coverage that occurred without. Cells were combined in the ratio of 30% HBMEC, 15% pHBVP, 15% HA, 5% HM, 15% HO, and 20% HN. First, neuronal‐glial organoids containing HA, HM, HO, and HN were allowed to preform for 48 h before subsequent inclusion of rat tail collagen I, HBMEC and pHBVP to grow an encapsulating layer about the preformed neural–glial organoid. Individual 6‐cell organoids were matured for a further 48 h in 96 well plates and remained viable for at least 6–10 days.^[^ ^351^ ^]^

These organoids expressed transcellular markers (P‐gp and GLUT‐1) as well as paracellular markers (of TJs: ZO‐1 and claudin‐5, and adherens markers: VE‐cadherin and beta‐catenin). Furthermore, the utility of the 2‐stage, 6‐cell organoids to toxicological and pathological models was validated in the following three scenarios:^[^ ^351^ ^]^

- Permeabilization response to hypoxia of endothelia‐enveloped organoids, modeling stroke pathology.
- Resistance of endothelia‐enveloped organoids to Hg2+ toxicity, modeling heavy metal poisoning.
- Susceptibility and resistance of endothelia‐enveloped organoids to MPTP/MPP+ toxicity, modeling neurotoxin‐induced Parkinson's disease.

#### 5.2.2 Permeabilization of In Vitro Organoid BBB by Hypoxia, Modeling Stroke

Hypoxia is a driver of neurological injury associated with stroke, and has been shown to induce permeability changes in a transwell model of cerebral BBB.^[^ ^352^ ^]^ Exposure of 6‐cell organoids to 24 h hypoxia (0.1% O_2_) at day 6 of culture appeared to disrupt the expression of markers of paracellular barrier function (claudin‐5, ZO‐1, beta‐catenin and VE‐cadherin) and was confirmed to bring about a statistically significant reduction in relative staining of two (beta‐catenin and VE‐cadherin). This organoid model may therefore be of use in modeling the pathology of response to barrier disruption during stroke.

#### 5.2.3 Selective Movement of Inorganic Mercury across In Vitro Organoid BBB, Modeling the Neurotoxicity of Heavy Metal Poisoning

Antioxidant systems of the brain depend upon selenoenzymes. Mercury poisoning involves their irreversible inhibition by sequestration of selenium. Because of their positive charge, mercury II salts are hydrophilic, highly soluble and do not cross the intact paracellular barrier. The susceptibility of glia and neurones of spheroids to mercury toxicity was used to validate the charge selectivity of the barrier function of covering endothelia.^[^ ^351^ ^]^ Barrier functions of organoids made entirely from neurones, therefore lacking an endothelial barrier layer (BBB^−^), were compared with 6‐cell stage‐assembled organoids having an endothelial barrier (BBB^+^). Assessment of their susceptibility to poisoning by mercury II chloride was made using ATP production as a measure of cell viability. Consistent with a lowered permeability of Hg^2+^ through an intact endothelial barrier (BBB^+^), neuronal organoids that lacked an endothelial barrier (BBB^−^) demonstrated significantly higher cell death, measured by reduced ATP production. That ATP production significantly increased in BBB^+^ organoids exposed to mercury II chloride, is consistent with elevated demand for Na‐K‐ATPase activity^[^ ^353^ ^]^ to sustain facilitated export of ions. That ATP production in BBB^+^ organoids exposed to mercury II chloride was dependent on barrier activity was confirmed by permeabilizing with histamine, which lowered ATP production, indicating penetration of mercury.

#### 5.2.4 Neurotoxicity of MPTP in 6‐Cell, Stage‐Assembled Organoids

In vivo, the lipophilic small molecule prodrug 1‐methyl‐4‐pheynl‐1,2,3,6‐tetrahydropyridine (MPTP) is known to be enzymatically converted by monoaminoxidase‐B (MOAB) of glial cells to form the neurotoxic metabolite 1‐methyl‐4‐phenylpyridinium (MPP^+^), which depletes dopaminergic neurones to model drug‐induced PD. In the in vitro BBB+ model, MPTP was shown to cross the 6‐cell, stage‐assembled organoids due to its lipophilicity, was converted to MPP^+^ by glia and caused a reduction in ATP production/viability.^[^ ^351^ ^]^ In contrast, ATP production of neuronal organoids lacking the endothelial envelope (BBB^−^) were unaffected. Conversely, MPP^+^ was toxic to BBB^−^ neuronal organoids, reducing ATP production, but BBB^+^ organoids were impervious to the hydrophilic compound. The selective resistance to MPP^+^ of BBB^+^ organoids was dependent upon barrier activity. This was demonstrated by the permeabilization of endothelial barrier in response to histamine treatment, which caused a significant fall in ATP production/viability. Taken together, these results confirm the utility of in vitro 6‐cell, stage‐assembled organoids in modeling relevant pathological and toxicological challenges to the BBB.

### 5.3 Dynamic In Vitro Models—Microfluidic Vascular Networks

DIV models of the BBB achieve media flow through an artificial capillary‐like structure.^[^ ^325^, ^327^, ^354^ ^]^ Hollow fibers incorporating channels and made from gas‐permeable silicone tubing are used to create a frame onto which cocultures are seeded and grown: Intraluminal surfaces are lined by ECs; accessory cells (pericytes and astrocytes) are grown abluminally.^[^ ^327^, ^354^ ^]^ The self‐assembly of mixtures of cells on flat, optically accessible forms, perfused by the peristaltic pumping of media recreates vasculogenic conditions, which result in networks of patent circular vessels resembling capillaries in vitro. Physiologically relevant pulsatile pumping of media replicates circulatory activity in the formation and maintenance of vessel architectures and barrier integrity.^[^ ^325^ ^]^ DIV models thereby provide not only the cellular interactions of ECs with accessory cells (particularly PEs and astrocytes) but also the shear force derived from fluid flows. These are two important stimuli known independently to maintain barrier phenotype.

Campisi et al. recently created the first capillary‐like vascular structures by self‐assembly incorporating three human cell types (iPSC‐EC combined with primary brain pericytes and astrocytes) cocultured in a 3D cast created using a polydimethylsiloxane (PDMS; Sylgard) based soft lithography technique.^[^ ^325^ ^]^ The chip was accessible to confocal fluorescence assay, while lysis of cells was achievable for biochemical/expression assay. The geometric architecture of the micro‐vasculature networks obtained was visible under glass and characterized by confocal imaging in terms of a) lateral and transverse vessel diameters, b) percentage of image area containing vessels, and c) total branch length. Direct physical contacts of pericytes and astrocytes developed in vitro, resembling the in vivo situation. Contact with pericytes or the conditioned medium of pericytes promoted smaller, more rounded and highly branched vessels than iPSC‐ECs in monoculture, and this interconnected and branched architecture was further enhanced by tri‐culture with astrocytes. While the overall transverse diameters were similar in all conditions (≈30 µm) lateral vessel diameters were successively reduced compared with iPSC‐ECs in monoculture (108 µm) by co‐ (64 µm) and tri‐culture (42 µm); tending toward greater circularity in tri‐culture compared with more elliptical vessel shapes in monoculture. Likewise, average branch length decreased from monoculture (266 ± 40 µm) to coculture (179 ± 31 µm) to tri‐culture (136 ± 24 µm). Vascular networks were more connected, narrower and covered less area, more similar to in vitro vessel morphology.^[^ ^325^ ^]^

Consistent with improved barrier functions in the tri‐cultures, immunocytochemical and mRNA expressions of ECM proteins (laminin and collagen IV) and junction proteins (ZO‐1, occludin and caudin‐5) were increased. Permeability coefficients were measured by introducing fluorescently labeled dextran tracers and capturing confocal images at 5 min intervals. The permeability of 40 kDa FITC‐dextran decreased progressively under mono‐, co and tri‐culture (6.6; 2.5 and 0.89 × 10^−7^ cm s^−1^, respectively). A similar trend was observed with 10 kDa FITC‐dextran. Moreover, permeabilities of this model were reportedly lower than other previously published microfluidic models.^[^ ^325^ ^]^

The resemblance to in vitro properties of microvascular assemblies derived from this method compared with static models was suggested. Various applications are proposed for such microfluidic models, including not only the assay of BBB permeability to fluorescently labeled therapeutic nanocarriers,^[^ ^354^ ^]^ but also the investigation of vascular interaction with inflammatory cells that occurs in neurodegenerative disease, or metastatic cancer extravasation in this promising model.

### 5.4 Comparing In vitro BBB Methods

In vitro simulations of the BBB aim to inform the potential for pharmaceutical success of drug candidates for neurological disorders. The same models also offer a means to advance our understanding of physiological mechanisms and pathological processes of the brain.

Current in vitro methods are far from a perfect facsimile of the BBB in vivo, but our review illustrates ongoing advances in alternative techniques. The most widely used model (endothelia as monolayers on transwell inserts) is notable for the ease with which luminal and abluminal compartments can be sampled, that TEER can be measured, and the facility with which high paracellular barrier properties from experimental animal (e.g., porcine) monolayers can be obtained. While the derivation of cells from renewable sources (hiPSCs), grown in RA, without VEGF‐A supplementation and in coculture with associated cells (astrocytes, pericytes, glia and neuronal cells) are each ways to improve the paracellular barrier properties and the expressions of transcellular transporters of human in vitro BBB models.^[^ ^342^ ^]^ Moreover, cocultures serve to mimic not only the pharmacokinetic properties of a) endothelial permeability but also b) the fraction of drug bound to brain tissue (*f*_u,br_) and as such give better correlations between transwell in vitro models and in vivo data on the permeation and tissue binding of a range of drug types.^[^ ^335^ ^]^

Although less established, in vitro spheroid BBB models are simple and reliably grown. The use of hiPSC derived cells for self‐assembly into minimally the 3‐cell organoids, or ideally from 6‐cells known to be involved in the NVU. Nzou et al. provides a renewable model, where the direct contact of cell types present in vitro is recapitulated.^[^ ^351^ ^]^ Moreover, organoids simulate endothelial permeability as well as drug binding to brain tissue. Because organoids are conglomerates of cells, they do not have the same facility of transwell for sampling of luminal and abluminal compartments or measuring TEER. However, fluorescently labeled drugs may be followed in spheroids using confocal slice imaging (readily available), else resolution of unlabeled drugs may be resolved to 30 µm within frozen sections by MALDI‐MSI^[^ ^347^ ^]^ if the technique is available. Spheroids better model the direct physical association of cells, albeit in an “inside‐out” topology of the NVU, bettered in this respect only by more complex and less widely used fluidic “capillary‐like” models. Already, spheroids have been rated favorably when compared with transwell models,^[^ ^347^ ^]^ and are proposed as the next generation of high throughput screening for BBB‐penetrant drug candidates, as well as to model BBB properties associated with stroke, degenerative or toxicological neurological disorders.^[^ ^351^ ^]^

Developments in DIV models have benefited significantly from technological advances in printing manufacture, which have supported reduction in vessel size, a network complexity resembling that in vivo and achieving microfluidic devices accessible to optical and molecular assay.^[^ ^354^ ^]^ There is also potential to incorporate fluid flow into versions of existing static models, particularly for spheroids. However, fluidic methods are not as widespread or established as to yet obtain the convenience of transwell, which are readily available as commercial kits (e.g., RBT‐24H, BBB Kit).^[^ ^355^ ^]^ **Table** **6** summarizes the pros and cons of the different presented BBB models.

**Table 6:** Comparative features of in vitro models different BBB models

Preclinical development of CNS drug targets in human diseases involves complementary in vitro modeling methods. Summary advantages and disadvantages of variants of these models of the BBB are herein reviewed.		
	Advantages	Disadvantages
In vivo	+ Animal models are considered the gold standard of preclinical predictive tools, but ≈80% of drug candidates identified in this way later fail in clinical trial	– Animal models are analogous in many of the cell, molecular, organ system functions and connections found in man, but may not accurately reflect the human condition – Relatively low throughput, expensive, and limited by ethical considerations
In vitro: static (transwell and organoid)		
	+ Includes the barrier phenotypes induced by cellular contacts of EC with a basement membrane, each other, and if included in coculture various other cells of the NVU, notably pericytes/vascular smooth muscle cells and astrocytes	– Omits the barrier phenotypes that are induced and maintained by the shear forces imparted by circulating plasma on ECs
Transwell	A suspended semi‐permeable platform on which endothelial monolayer grown; with or without cocultured cells on abluminal surface or on base of well into which insert placed	
Monolayer (transwell)	+ Commonly used (body of comparative work is available) + Convenience of treatment and sampling of luminal and abluminal compartments; ease of tissue sourcing + Robust barrier properties of animal (porcine or bovine) brain EC monolayers are an impressive option	– Human monolayers have poor barrier properties by comparison – Omit the cellular contacts made by EC with other cells of the NVU, notably pericytes/vascular smooth muscle cells and astrocytes that determine barrier phenotype – Omits the barrier phenotypes that are induced and maintained by the shear forces imparted by circulating plasma on ECs
CO‐/multi‐culture (transwell)	+ Can achieve good correlation between in vitro and in vivo drug permeability, validating predictive value, but cellular composition critical: Presence of cocultured glial cells in the abluminal compartment better mimicked the effect of brain tissue binding that occurs in vivo	– Omits direct cellular contacts that induce barrier properties (that are present in 3D spheroid organoids or microfluidic organoids); also, does not mimic shear forces of fluidic systems
Multi‐culture variants (primary, immortalized, and renewable stem cells) Features relevant to all in vitro platforms—transwell, organoid, and organ‐on‐a‐chip		
Primary cells	+ Differentiated phenotype; clinically relevant if human	– Difficult to source human cells. – Brief use (limited growth)
Immortalized (cell lines)	+ Convenience of renewable cells; of which commercial banks available	– Lack of polarization–differentiation state might not reflect normal – Generally, well characterized, but susceptible to genetic changes over multiple passages
Renewable stem cells	+ Renewable, sustainably available (cells can be frozen/grown up) + Human cells are “induced,” not requiring placental material + Can avoid immunogenic mismatch if all cells derived from same progenitor + Self‐aggregating, thought to recapitulate cues for development of barrier phenotype	– Requires technical expertise; complexity; delay in induction of phenotypes
Spheroid/organoid Multicellular, self‐assemblage of brain cells enveloped in endothelia	+ Relatively new model, but now established and becoming validated + Available in variants of complexity, using primary, immortalized or “renewable” stem (isolated or induced) human cells + Includes the barrier phenotypes induced by direct cellular contacts	– Omits the barrier phenotypes induced and maintained by shear forces
Dynamic in vitro models (“organ‐on‐a‐chip”)	+ The fullest representation of the complex cellular and shear force interactions that establish and sustain barrier properties	– Relatively low throughput
In silico	+/‐ Models involve computation based on physiochemical and physiological parameters derived from/requiring validation by in vivo/in vitro studies	+/‐ Provide leads; need validation

## 6 Interactions of Nanocarriers with the Complement System

Investigators should evaluate the risks and benefits of the formulations before starting clinical trials. A clinical trial may be justified when the benefits of a formulation are greater than the risks.^[^ ^356^ ^]^ Most of the brain‐targeting nanoparticle formulations are designed to be injected intravenously. Therefore, these formulations should show blood compatibility, nontoxicity, and no immunogenicity to minimize risks to the patients. Consequently, Sections 6 and 7 cover these aspects of the formulations.

**Figure 11:**
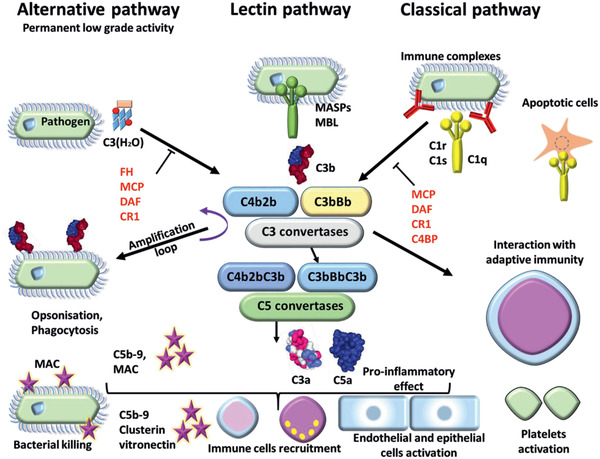
Complement activation. Complement system is composed of three different pathways. The classical pathway (CP) is activated by immune complex formation on pathogen surface and by calreticulin expressed on apoptotic cells, leading to C1 complex association. The LP recognizes mannose‐terminating glycan on pathogens leading to MBL MASP complex activation. Both induce formation of the classical C3 convertases C4b2b. The AP is permanently activated at low level by spontaneous hydrolysis of C3 into C3 (H_2_O). Lack of complement inhibitor on pathogens induces alternative C3 convertase activation C3bBb. Complement activation leads to opsonization and phagocytosis by C3b deposition, bacterial lysis by C5b–9 complex formation and inflammation by recruitment of immune cells, endothelial and epithelial cell activation, and platelet activation. Membrane cofactor protein (MCP), decay accelerating factor (DAF), complement receptor 1 (CR1), and C4 binding protein (C4BP) inhibit the complement activation by the classical pathway. DAF, MCP, CR1, and Factor H inhibit the complement activation by the AP.

The complement system is one of the most critical components of innate immune responses that have evolved to protect the host from invading pathogens like bacteria and viruses, and also to eliminate any foreign materials that enter the body.^[^ ^357^, ^358^ ^]^ The complement system has the ability to distinguish “self” from “nonself”/“foreign” material and to eliminate or neutralize them. Contact with a “foreign” material or an invading pathogen results in the activation of the complement system. Depending on the nature of the trigger, the ensuing complement activation may cascade via one of the three distinct pathways namely a) the classical pathway, b) the alternative pathway (AP), and c) the lectin pathway (LP) (**Figure** **11**).

The classical pathway is activated by immune complexes that are formed on pathogen surfaces leading to C1 complex formation while the mannan‐binding lectin (MBL) pathway recognizes mannose‐terminating glycans on pathogens.^[^ ^357^ ^]^ The binding of MBL to carbohydrates on the pathogen surface activates MBL‐associated serine proteases (MASPs).^[^ ^359^ ^]^ The MBL‐MASP complexes activate C3 directly or generate C3 convertase. Essentially, the activation of both classical and LPs result in the formation of highly reactive C3b that binds to hydroxyls and amines on foreign surfaces forming a membrane pore complex C5b‐9 and potent anaphylatoxins C3a and C5a.^[^ ^360^ ^]^ In the AP, a permanent low‐level activation of C3 by spontaneous hydrolysis of a thiolester leads to production of C3 (H_2_O).

Due to the lack of natural complement regulators factor H (FH) and factor I on pathogen surface, alternative activation of C3 convertase C3bBb occurs. C3 convertase cleaves C3 into C3a (anaphylatoxins) and C3b (opsonin). Complement activation leads to a multitude of responses that include deposition of C3 fragments (C3b) on the surface of the pathogen (opsonization) which in turn leads to phagocytosis, formation of the membrane‐damaging C5b‐9 complex which effectively induces bacterial lysis, release of complement peptides C3a, C4a and C5a (referred to as anaphylatoxins) that mediate recruitment of immune cells resulting in inflammation and activation of epithelial, endothelial cells and platelets.^[^ ^357^, ^361^ ^]^ Uncontrolled release of anaphylatoxins may promote life threatening inflammatory reactions that may lead to severe cardio‐respiratory distress and even cardiac arrest and death.^[^ ^362^ ^]^

### 6.1 Nanoparticles and the Complement System

Because NCs are largely man‐made synthetic structures, they are typically recognized by the host immune system as “foreign” and evoke complement activation. NP‐induced complement activation has been reported with a variety of NCs including gold and silver NPs,^[^ ^19^ ^]^ SPIONs,^[^ ^363^ ^]^ polymer NCs,^[^ ^20^ ^]^ liposomes,^[^ ^21^ ^]^ micelles,^[^ ^364^ ^]^ CNTs,^[^ ^365^ ^]^ graphene,^[^ ^366^ ^]^ and dendrimers.^[^ ^367^ ^]^ In the following sections the activation of the complement system is discussed when NPs are administered i.v., however, the brain also has the complement system, which can be activated by nanosize objects such as A*β* plaques.^[^ ^368^ ^]^ This leads to attraction and activation of glial cells, which under such activation conditions, can produce neurotoxic substances. Therefore, the complement activation also should be taken into consideration, when NPs are delivered intracerebrally. Then in this article, it is presumed NPs that activate the complement system in the blood, would also activate the complement system in the brain.

### 6.2 Nanoparticle‐Induced Complement Activation‐Related Pseudoallergy (CARPA)

Activation of immune cells with the consequent production of anaphylatoxins and cytokines can sometimes result in severe and immediate hypersensitivity reactions with symptoms and signs of anaphylaxis such as chills, rigors, facial flushing, facial swelling, headaches and cardiorespiratory distress.^[^ ^369^ ^]^ Since this reaction is not a true IgE mediated hypersensitivity reaction, Szebeni coined the term Complement Activation Related Pseudoallergy or CARPA to distinguish it from the Type I IgE‐mediated hypersensitivity reactions.^[^ ^369^ ^]^ CARPA is a well‐documented adverse reaction associated with a variety of nanopharmaceuticals including Doxil (liposomal doxorubicin), Ambisome (liposomal amphotericin), Taxol (cremophore‐paclitaxel), Sandimmune (cremophore‐cyclosporine A) and Ferridex (SPIONs).^[^ ^369^, ^370^, ^371^, ^372^ ^]^ The pathogenesis of CARPA is thought to be due to the anaphylatoxins (C3a and C5a) and other C3 fragments such as C3d.^[^ ^373^ ^]^ The generated anaphylatoxins bind to their receptors on the immune cells such as macrophages, mast cells and basophils and trigger the release of various vasoactive substances including histamine, leukotrienes, platelet activating factor, prostaglandins and thromboxane A_2_. Although CARPA has proved fatal in some cases, it can often be treated with antihistamines, corticosteroids and epinephrine.^[^ ^374^ ^]^ It may also be possible to prevent CARPA by the pre‐administration of complement inhibitors such as indomethacin or desensitization using empty liposomes.^[^ ^375^ ^]^

### 6.3 Complement‐Mediated Clearance of Nanoparticles

The complement system plays a major role in clearing nanomaterials from the circulation. As outlined in the preceding sections, NPs, upon entering the circulation, activate the complement system. The activation of the complement system leads to the generation and deposition of opsonins such as C3b on the surface of the NPs (opsonization). The opsonized NPs are rapidly engulfed by the cells of the RES (e.g., macrophages) that express complement receptors.

In addition, NPs can also be recognized and taken up by various blood cells. Neutrophils express complement receptors C1qR, CR1, CR3, and CR4 that are involved in phagocytosis of bacteria,^[^ ^376^ ^]^ while lymphocytes express complement receptors CR2 and CR1.^[^ ^377^ ^]^ Monocytes are known to uptake NPs. Platelets have been shown to bind to C3b via glycoprotein IIb/IIIa.^[^ ^378^ ^]^ Leroux et al. demonstrated that poly(d,l‐lactic acid) NPs are internalized by monocytes and macrophages.^[^ ^379^ ^]^ Inturi et al. have shown that iron oxide NPs are recognized by leucocytes and platelets via complement C3 and engulfed. They have also shown that the uptake of iron oxide NPs by leucocytes can be prevented by inhibitors of the AP of the complement system such as antiproperdin antibody.^[^ ^380^ ^]^ The role of neutrophils in the uptake of biodegradable NPs was demonstrated by Zambaux et al.^[^ ^381^ ^]^

### 6.4 Influence of Nanoparticle Characteristics on Nanoparticle‐Induced Complement Activation

Several intrinsic properties of nanomaterials such as the composition, size, shape, surface charge and surface characteristics/modifications, are known to influence the nature and the magnitude of NP induced complement activation.

#### 6.4.1 Nanomaterial Composition

The impact of nanomaterial composition on the complement activation is particularly noticeable in lipid NCs (e.g., liposomes, micelles) and polymer NPs. For instance, the chain length and architecture (e.g., linear or branched) of polymer NPs can affect the interaction between the nanomaterial and the complement system. Furthermore, the chemical make‐up of the polymer (e.g., hydrophobicity/hydrophilicity) and the types of functional groups also influence complement activation.^[^ ^382^ ^]^ Hulander et al. studied the potential of various noble metal NPs and Bactiguard, which is a composite material, to generate complement factor 3 fragment (C3a). Their study showed that the C3a generation potential varied among the noble metal NPs (silver>gold>palladium>Bactiguard>titanium).^[^ ^383^ ^]^ Wibroe et al. examined the effect of graphene oxide (GO) form (in solution versus immobilized) and oxidation state on the complement system in human blood. In solution, there was a decrease in GO‐mediated complement activation with decreasing surface oxygen content, whereas with immobilized GO complement response was reversed and increased with decreasing oxygen content.^[^ ^366^ ^]^

#### 6.4.2 Particle Size

The size of NPs plays an important role in complement activation. Pedersen et al. investigated the importance of the curvature of the NP in complement activation using dextran‐coated NPs of different sizes (250 and 600 nm) and human IgM antibodies to dextran. Interestingly, potent antibody‐mediated complement activation was noted on 250 nm sized NPs whereas 600 nm particles were less potent activators.^[^ ^384^ ^]^

Pham et al. investigated the effect of particle characteristics including the size and polydispersity on nanomaterial‐induced complement activation using a metric called “Residual Hemolytic Activity” (RHA) for perfluorocarbon NPs of varying size, charge and surface chemistry. Subsequently, the researchers analyzed the above data using a decision tree learning algorithm to determine the effect of certain NP characteristics on complement activation.^[^ ^385^ ^]^ The authors claim that physicochemical properties of NPs (size, zeta potential, and polydispersity index (PdI)) can serve as good predictors of NP‐dependent complement activation.^[^ ^386^ ^]^ This study predicted that reducing the particle size would reduce the chance of complement activation. For example, NPs with the size of 204.55 nm were low complement activator (being safe), while increasing the particle size to 259.4 nm (maintaining the same zeta potential) turned the NPs to a highly activating complement formulation.

#### 6.4.3 Surface Charge

Surface charge has been shown to play a role in NP‐mediated complement activation. Several studies have shown that charged nanomaterials are more efficient activators of the complement system compared to their neutral counterparts.^[^ ^387^ ^]^ This observation has been made with a variety of nanomaterials including polypropylene sulfide NPs, lipid nanocapsules, cyclodextrin‐containing polycation based NPs and polystyrene nanospheres. Both positively charged and negatively charged NPs have been shown to activate the complement system. The study carried out by Szebeni and co‐workers investigated CARPA in a porcine model and demonstrated that high negative surface charge plays a critical role in the causation of this serious adverse reaction associated with several liposomal nanopharmaceuticals.^[^ ^372^ ^]^ However, using polymer NPs, Mayer et al. showed that positive surface charge induced activation of complement too.^[^ ^388^ ^]^

Misra et al. undertook a study to determine the effects of various NP characteristics (size, surface charge, molecular chemistry, and molecular weight of coating agents) on complement activation using carbon spherical NPs. Their study showed that the presence of a highly positive surface charge enhanced complement activation.^[^ ^389^ ^]^ Methoxy polyethylene glycol grafted liposomes are known to evoke immediate non‐IgE mediated hypersensitivity reactions. The complement activation is thought to be due to the presence of anionic phosphate‐oxygen moiety of the PEGylated phospholipid.^[^ ^390^ ^]^ Interestingly, Moghimi et al. showed that methylation of this phosphate oxygen moiety prevents PEGylated liposome mediated complement activation.

#### 6.4.4 Polydispersity

NP aggregates are known to induce complement activation. In a study carried out by Mayer et al. to investigate the effect of size and surface charge of the NPs on complement activation, induction of coagulation and hemolysis and activation of granulocytes and platelets, it was shown that positive charge induced formation of NP aggregates and complement activation.^[^ ^388^ ^]^ A similar observation was made in a recent study undertaken by Fülöp et al. to study the role of iron core composition and particle surface coating in six different commercially available superparamagnetic iron oxide NPs. This revealed that Sinerem is a strong activator of complement. NP tracking analysis of the particles showed that Sinerem displayed a multimodal size distribution including a significant fraction of aggregates strengthening the supposition that NP aggregates can lead to complement activation and CARPA.^[^ ^391^ ^]^

#### 6.4.5 Surface Characteristics/Modifications

Surface characteristics, both the intrinsic chemical composition of the nanomaterial and any introduced modifications (“functionalized”) play a pivotal role in complement activation. Of the intrinsic chemical structures that are well known to induce complement activation are the repetitive sequences and high surface density of amino and hydroxyl moieties. Amino and hydroxyl groups are capable of directly activating and accelerating AP.^[^ ^392^, ^393^ ^]^

A widely used strategy to reduce NP clearance from circulation by the RES is to decorate the particle surface with certain molecules such as PEG, dextran or other polymeric coatings. This strategy has proved effective in clinical pharmacology in that this modification has significantly improved the pharmacokinetic properties including prolonged circulation time of a number of pharmacological agents.^[^ ^394^, ^395^, ^396^, ^397^, ^398^, ^399^, ^400^, ^401^ ^]^ However, published reports also suggest that there is a small risk that this process may induce complement activation which in turn may lead to anaphylactic reaction.^[^ ^401^, ^402^, ^403^, ^404^ ^]^ Both PEG and dextran have been reported to cause CARPA. So much so that, several dextran‐coated SPION preparations (Sinerem, Combidex, and Ferridex) that have been used in imaging as contrast medium were withdrawn from the market. For this reason, dextran‐coated NPs have come under intense research scrutiny in recent years.

#### 6.4.6 Dextran‐Coated Nanoparticles

Dextran is a polysaccharide and as such it can activate the complement system via the LP.^[^ ^363^ ^]^ However, it can also trigger the complement system in human blood through the AP.^[^ ^405^ ^]^ It should be noted that the potential to induce complement activation varies significantly depending on the chemical composition of the dextran compound used. In order to explore the roles of iron core composition and particle surface coating in SPION‐induced CARPA, Fulop et al. measured complement activation by six different SPIONs in a human serum that is known to react to NPs with strong complement activation. Interestingly, only the carboxymethyldextran‐coated (ferucarbotran, Resosvist) and dextran‐coated (ferumoxtran‐10, Sinerem) SPIONs caused significant complement activation, while the citric acid, phosphatidylcholine, starch and chitosan‐coated SPIONs had no such effect.^[^ ^391^ ^]^

In addition to its composition, the configuration of the dextran layer also plays an important role in complement activation. Bertholon et al. investigated the influence of surface morphology, length, and type of polysaccharide on complement activation by measuring the degree of conversion of C3 into C3b in serum incubated with NPs. Their study showed that the complement activation induced by dextran‐coated NPs increased with the size of dextran bound in “loops” compared to that of “brush” configuration. The authors suggested that the observed difference could be explained by an increasing steric repulsive effect of the brush, inducing poor accessibility to OH groups.^[^ ^406^ ^]^

There appears to be a species difference in the mechanism of complement activation caused by dextran‐coated SPION. Banda et al. carried out a study to investigate the mechanisms of complement activation in human and mice using superparamagnetic iron oxide nanowires (SPIONW) showed that the complement activation in mice was mediated via the LP while direct enhancement of the AP was the main mode of activation in human serum.^[^ ^363^ ^]^

In order to minimize dextran mediated complement activation, Unterweger and colleagues made modifications to the composition of the dextran coating. Magnetic NPs were coated with a formulation containing low molecular weight (10 kDa) unmodified dextran, mannitol and citrate. Hemocompatibility (effects on red cells, leucocytes, platelets, coagulation, and complement system) of these particles (named SPIONdex) were studied in vitro. In addition, the researchers conducted in vivo experiments using domestic pigs to investigate its potential to induce CARPA following i.v. infusion. The researchers report that SPIONdex is highly hemocompatible with minimal adverse effects on blood cells, coagulation, and complement system. Significantly, SPIONdex particles did not induce CARPA even at high concentration.^[^ ^407^ ^]^

#### 6.4.7 Polyethylene Glycol Coated Nanoparticles

PEG, a highly hydrophilic polymer, is the most widely used stealth polymer for NCs in order to reduce clearance by the RES thus extending circulation time and improving drug efficacy.^[^ ^408^ ^]^ However, polyethylene glycol coated (“PEGylated”) pharmaceutical formulations such as Doxil (PEGylated liposomal doxorubicin) have been associated with severe hypersensitivity reactions.^[^ ^409^ ^]^

Several reports indicate that antibodies to PEG play a direct role in inducing complement activation via antibody‐mediated classic pathway.^[^ ^408^, ^410^ ^]^ Anti‐PEG antibodies have been shown to be present in the blood of patients who have been exposed to PEGylated pharmaceuticals^[^ ^411^ ^]^ and also in unexposed population.^[^ ^412^, ^413^, ^414^ ^]^ In addition to inducing hypersensitivity reactions, these antibodies also cause accelerated clearance of PEGylated drugs from blood leading to reduced clinical efficacy.^[^ ^414^, ^415^ ^]^

Although activation via the classic pathway appears to play a major role in PEGylated NCs induced complement activation, other pathways (alternate and lectin) may also play a role. Hamad et al. studied the interaction between PEGylated SWCNTs and the complement system. They showed that PEGylated SWCNTs activated complement and induced production of C4 without any increase in alternate pathway activation. Furthermore, SWCNT induced complement activation was unaffected by depletion of C1q with anti‐C1q antibody but not antimannan‐binding lectin serine protease (MASP) 2 antibodies. The authors suggested a possible LP in PEGylated SWCNTs induced complement activation.^[^ ^416^ ^]^

### 6.5 Nanoparticle‐Induced Complement Activation: The Challenge for Brain Drug Delivery

Despite the tremendous advances made over the last few years in understanding the pathogenesis of NP‐induced complement activation and the associated clinical syndromes, hitherto no reliable solutions to overcome this potentially serious complication of NCs have been discovered. Hence, development of effective measures to minimize or eliminate this adverse reaction will be an essential prerequisite for successful NP‐based drug delivery. Although it remains to be determined the complement activation by conventional cell penetrating peptides such as RVG‐29, it has been suggested that the use of ApoA‐I may be advantageous in designing of brain targeting NPs.^[^ ^417^ ^]^ This is because ApoA‐I interacts with an activation‐dependent conformer(s) of the C9 component of the C5b‐9, which inhibits C9 polymerization, hence minimizing the risk of complement activation.^[^ ^418–365^ ^]^ On the other hand, polystyrene NPs form a protein corona in serum with ApoA‐I being one of the dominant adsorbed proteins.^[^ ^420^ ^]^ However, this does not prevent complement activation by polystyrene NPs in human serum.^[^ ^421^ ^]^ Therefore, further investigations are required to examine the suitability of using ApoA‐I as a brain targeting ligand for NPs and avoiding complement activation by these NPs.

## 7 Cytotoxicity, Hemolysis, and Immunogenicity

When NPs/NCs are employed for drug delivery to the brain, these NPs/NCs should be nontoxic to bystanding cells as well as neurons, in particular when the cargo is noncytotoxic agent. Furthermore, when brain targeting NPs/NCs are administered i.v., these should not cause hemolysis as loss of red blood cells (RBCs) may lead to anemia. Finally, brain targeting NPs/NCs should be noninflammatory and nonimmunogenic. Otherwise, they may be cleared rapidly from the blood stream and brain by macrophages; or the NPs/NCs may activate brain microglia causing undesired injuries to brain cells, or worsen the damages to neurons in diseased brains. In the following sections, the toxicity of NPs is discussed as well as their immunogenicity and hemolysis.

### 7.1 Cytotoxicity

It is quite critical that NP formulations are assessed and confirmed to be safe in terms of their nontoxicity to cells, lack of (or negligible) capacity to provoke unwanted immunological reactions, and nonlytic effects on the RBCs. The NP formulations targeting the CNS are tested experimentally using neuronal cell lines and primary neurons in vitro to assess their cytotoxicity and immunogenicity.^[^ ^22^ ^]^ These neuronal cells are very useful in modeling various brain diseases through application to them of a wide range of experimental agents under several different experimental conditions. As a result of their versatility, they are an excellent tool in elucidating the cellular and molecular mechanisms underlying CNS disorders and are, therefore, important to the study of brain‐targeting NPs. A battery of assays can be used to assess the effects of a NP on cell (including neuronal) viability (or its induction of cell death), usually by quantifying treatment‐induced changes to the levels or activities of a specific enzyme or a group of enzymes, or residual cellular energy content as indicated by intracellular levels of adenosine triphosphate (ATP),^[^ ^422^, ^423^, ^424^ ^]^ or residual levels of some other intermediate for metabolic activity. These assays are commonly based on absorbance, fluorescence or luminescence readouts. Some of them are destructive (lytic) and are, therefore, only useful for endpoint determinations, while some are nondestructive and, therefore, able to assess changes in either endpoint or kinetic mode (time course), thus having an additional advantage of allowing the cells assayed for viability to be used for other assays, if required. Examples include the absorbance‐based tetrazolium reduction (3‐(4,5‐dimethylthiazol‐2‐yl)‐2, 5‐diphenyl tetrazolium bromide [MTT]) assay (one of the most commonly used) or any of its more recent improved versions (MTS, XTT, WST‐1); the alamarBlue assay (fluorescence‐ or absorbance‐based), and the bioluminescence‐based ATP assays.^[^ ^423^, ^425^, ^426^ ^]^

While the underlying assumption in using these assays is that the extent of the changes to the surrogate parameters being captured correlates well with metabolic activity and cell viability, each assay has caveats associated with it that must be taken into consideration while deciding which one(s) to employ. One of the major challenges is the potential interaction between a test substance and the assay reagents or systems, which can lead to false positives and, therefore, has to be properly controlled for in the design of viability testing experiments. In this context, it should be noted that some NPs have demonstrated, or are suspected to have, real potential for interacting with cell viability reagents and this reality or possibility should be considered when assessing cytotoxicity of NPs.^[^ ^427^, ^428^, ^429^, ^430^ ^]^ A useful and recommended approach is to use at least two cell viability assays that are based on different readouts to assess the effects of NPs on cell viability. Such assays could be designed to run as separate or multiplex systems. There should also be appropriate controls to assess the potential interactions between NPs and the assay reagent or system in the absence of cells. Although for a given test substance differences in its toxicity profiles obtained from separate assays might be related to its mechanism(s) of induction of cytotoxicity, such differences could also be an indicator of unwanted interactions or artefacts inherent in one assay compared to the other(s) and could, as a result, guide the subsequent choice of assays as well as results interpretation. It is also good practice to assess the neurotoxicity of a NP using a range of neuronal cell lines and primary neurones.

**Table** **7** summarizes the cytotoxicity of recent brain‐targeting NP formulations evaluated using the MTT assay.^[^ ^431^ ^]^ It can be seen that inclusion of the transactivator of transcription (TAT) peptide in the NPs significantly increased their cytotoxicity. Furthermore, PLGA NPs conjugated with lactoferrin did not show cytotoxicity at concentrations as high as 10 000 µg mL^−1^, while conjugating PLGA NPs with TAT peptide reduced safe concentration down to 20 µg mL^−1^. These observations indicate certain brain‐targeting ligands may make NPs less cytotoxic compared to other brain‐targeting ligands. Interestingly, targeted exosomes showed a safe concentration limit of 20 µg mL^−1^, which is lower than for lactoferrin‐PLGA, RVG‐PLGA, and solid lipid NPs. Also, while RVG‐PLGA NPs did not show cell toxicity at a concentration of 1000 µg mL^−1^, the noncytotoxic concentration for RVG‐trimethylated chitosan NPs was 100 µg mL^−1^. Therefore, the NP core plays a crucial role in the cytotoxicity of the decorated NP with brain‐targeting ligand. As expected, loading NPs with cytotoxic chemicals increased the toxicity of the NP formulation.

**Table 7:** The cytotoxicity of NPs designed for brain delivery toward cell lines evaluated by the MTT test

NP formulation	90% Viability control NPs [µg mL^−1^]	Drug	90% viability loaded NPs [µg mL^−1^]	Cell line	Size [nm]	Ref.
Lactoferrin‐PLGA	10 000	Huperzine A	100	16HBE	153.2 ± 13.7	^[^ ^432^ ^]^
SLNP (cetyl palmitate)	1500	Functionalized with Apo E	1500	hCMEC/D3	192 ± 13	^[^ ^433^ ^]^
PLGA‐Tf	1000	Oxytocin	1000	RAW 246.7	197.7–278.3	^[^ ^434^ ^]^
PLGA‐RVG	1000	Oxytocin	1000	RAW 246.7	201.20 ± 3.35	^[^ ^434^ ^]^
Angiopep‐2 linked lanthanide‐doped upconversion NPs	300	IR‐780 + 5,10,15,20‐tetrakis(3‐hydroxyphenyl) chlorine	300 (no irradiation)	ALTSICI	80 ± 1	^[^ ^435^ ^]^
RVG‐trimethylated chitosan NPs	100	siRNA	100	Neuro‐2a	135 ± 7	^[^ ^436^ ^]^
PLGA	100	Diazepam	50	Green monkey kidney epithelial cells	190 ± 0.5	^[^ ^437^ ^]^
Mn‐LDH^a)^	100	siRNA	21.6	Neura2a	125	^[^ ^438^ ^]^
Magnetic poly (d,l‐lactide‐*co*‐glycolide) lipid	80	Glutathione	80	bEnd.3	102.0 ± 0.7	^[^ ^439^ ^]^
Poly (butylcyanoacrylate)	60	Cisplatin	12.5	A172	451.2 ± 11.1	^[^ ^440^ ^]^
SLNP (glyceryl distearate)	40	Asiatic acid	18	SVG P12 human fetal glial	94–141	^[^ ^441^ ^]^
*Acorus calamus* Ag (silver)	25	NA	NA	SH‐SY5Y	31.83	^[^ ^442^ ^]^
Magnetic poly (d,l‐lactide‐*co*‐glycolide) lipid‐TAT	20	Glutathione	<20	bEnd.3	131.8 ± 11.2	^[^ ^439^ ^]^
Targeted exosomes	20	Doxorubicin	<20^b)^	BT‐474	30–100	^[^ ^443^ ^]^
PLGA	2.5	Lamotrigine	1.25	Neuro‐2a	184.6	^[^ ^444^ ^]^
Liposomes	ND	1,9‐Pyrazoloanthrone	100	SH‐SY5Y	112.33 ± 0.84	^[^ ^445^ ^]^
gH625‐Fe_3_O_4_ magnetic NPs^c)^	ND	No drug but conjugating with gH625	20	HBMEC^d)^	104.0 ± 4.0	^[^ ^446^ ^]^

^a)^ Manganese based layered double hydroxide nanoparticles

^b)^ At 20 µg mL^−1^ the toxicity of exosomes was about 20% (cell viability 80%) similar to free Doxorubicin

^c)^ gH625 (H2N‐HGLASTLTRWAHYNALIRAFGGG‐CONH2)^[^ ^447^ ^]^ a BBB crossing peptide

^d)^ Human brain microvascular endothelial cells (HBMEC).

Lactate dehydrogenase (LDH) is an enzyme that is released as a result of cell membrane damage when cells are exposed to NPs. In the LDH assay the amounts of released LDH are quantified and are proportional to the extent of (necrotic) cell death.^[^ ^456^ ^]^ **Table** **8** presents a summary of typical NP formulations that were evaluated for cytotoxicity using the LDH assay. These NPs were formulated or considered for drug delivery to the brain. It can be seen that SWNTs present significant toxicity toward cells compared to the other formulations.

**Table 8:** The cytotoxicity of NPs designed for brain delivery toward cell lines evaluated by the Lactate dehydrogenase (LDH) assay

NP formulation	90% Viability control NPs [µg mL^−1^]	Drug	90% viability for loaded NPs [µg mL^−1^]	Cell line	Size [nm]	Ref.
SLNP (DSPE)	1850	Functionalized with Apo E3+resveratrol	3700	hCMEC/D3	167.8 ± 19.9	^[^ ^448^ ^]^
SLNP (cetyl palmitate)	1500	Functionalized with ApoE3	1500	hCMEC/D3	192 ± 13	^[^ ^433^ ^]^
G3 PAMAM	700	Lauryl chains and paclitaxel	35	Porcine brain endothelial cells	13.7 ± 1.3	^[^ ^449^ ^]^
Cationic (cetyltrimethylammonium bromide) SLNPs	33	NA	NA	Human neutrophils	195	^[^ ^450^ ^]^
zero‐valent iron	20	*α*‐Synuclein amyloid	<20	SH‐SY5Y	83.88^a)^	^[^ ^451^ ^]^
Single‐walled nanotubes	1	None	None	PC‐12	0.84 × 700–1000	^[^ ^452^ ^]^
Transferrin‐SLNP (cetyl palmitate)	ND	Quercetin	2400	hCMEC/D3	200	^[^ ^453^ ^]^
Nickel oxide	ND	NA	10	SH‐SY5Y	89.73 ± 5.98	^[^ ^454^ ^]^
Peptide nanofibers	ND	siRNA	11	hCMEC/D3	50 × 297	^[^ ^455^ ^]^

^a)^ This is hydrodynamic diameter, the actual NP diameter was 30 nm.

Cell toxicity induced by NCs or NPs could lead to a range of death outcomes, including apoptosis (programmed cell death) and necrosis. Knowledge of the potential of NCs or NPs to induce such death paradigms is, therefore, important, for example, in the case of NPs or NCs delivering noncytotoxic therapeutic agents such as nerve growth factor or siRNA to downregulate the BACE1 gene.^[^ ^23^, ^24^ ^]^Although apoptosis as a genetically programmed process is involved in organismal development and the maintenance of homeostasis, various external triggers are also known to initiate it. Apoptosis could be mediated through an intrinsic (mitochondrially mediated) pathway or an extrinsic pathway (via cell‐surface death receptors), and, depending on the nature of the toxic insult, could occur early or late in the death process.^[^ ^457^ ^]^ In early apoptosis, the cell membrane is intact, while in late apoptosis the cell membrane is permeable. Here we provide a few recent examples of the induction of apoptosis by brain‐targeting NPs. Dual functionalized (transferrin and penetratin) liposomes induced 10% apoptosis in bEnd.3 cells after 2 h of incubation,^[^ ^458^ ^]^ while poly (butylcyanoacrylate) NPs induced less than 1% apoptosis in A172 cancer cell line.^[^ ^440^ ^]^ Loading these NPs with anticancer drugs significantly increased their apoptotic effects. On the other hand, synthetic silicone dioxide NPs without any drug load induced apoptosis from 50 µg mL^−1^ concentrations and above in LN229 cell lines,^[^ ^459^ ^]^ whereas mesoporous silica NPs did not induce significant apoptosis in A127 cells at 374 µg mL^−1^.^[^ ^460^ ^]^ Interestingly, SPIONs did not induce apoptosis at 50 µg mL^−1^ in PC12 cells, but they promoted apoptosis at concentrations in the range of 60–200 µg mL^−1^.^[^ ^461^ ^]^

Necrosis is cell death characterized by cytoplasmic swelling and cells rounding up, bursting and collapsing, spilling out their intracellular content.^[^ ^462^, ^463^ ^]^ Leakage of cellular content into the extracellular medium represents a “danger signal” as it could provoke inflammation.^[^ ^464^ ^]^ Ag NPs caused necrosis at concentration in the region of 50 µg mL^−1^ in L‐929 fibroblast cells, with 10 nm NPs being more toxic than 200 nm NPs, causing more than 50% necrosis after 24 h incubation.^[^ ^25^ ^]^ Chiani et al. showed that blank PBCA NPs caused less than 1% necrosis, while loading these NPs with cisplatin did not increase necrosis in A172 brain cancer cell lines.^[^ ^440^ ^]^ On the other hand, biotinylated PAMAM G3 dendrimers substituted with the cyclooxygenase‐2 (COX‐2) inhibitor celecoxib and peroxisome proliferator‐activated receptor agonist (Fmoc‐l‐Leucine) caused late apoptosis (which was considered necrosis) in HaCaT cells at 4 × 10^−6^ m concentration.^[^ ^465^ ^]^ Interestingly, silica‐coated iron oxide NPs did not cause necrosis (or late apoptosis) at concentrations as high as 200 µg mL^−1^ in SH‐SY5Y cells.^[^ ^466^ ^]^

### 7.2 Hemolysis

The hemolysis test is conducted to evaluate the effects of NP formulations on the RBCs. Joshy et al. evaluated hemolysis of alginate–stearic acid/PEG NPs (ASNPs) on the RBCs. They reported no hemolysis or aggregation was observed in white blood cells, RBC and platelets.^[^ ^27^ ^]^ Ishak et al. tested lipid polymer hybrid NPs and gold NPs for hemolysis using rat blood (RBC) and then calculated percentage hemolysis by using the equation below. They reported all formulations were below permissible hemolysis threshold (5%) at 1 mg mL^−1^, whereas at 2 mg mL^−1^ the hemolysis was higher than the threshold^[^ ^467^ ^]^

(1) $$\mathrm{Hemolysis}\;\left(\%\right)=\;\frac{\mathrm{ODt}-\mathrm{ODnc}}{\mathrm{ODpc}-\mathrm{ODnc}}\;\times \;100$$

where ODt, ODnc, and ODpc are the optical densities of the test sample, the negative control, and the positive control, respectively. Hemolytic activity of brain targeting NPs/NCs have been investigated.^[^ ^458^, ^468^, ^469^, ^470^, ^471^, ^472^, ^473^, ^474^, ^475^, ^476^ ^]^ Among NPs or NCs for brain drug delivery, amphiphilic peptides (C16‐W‐I‐L‐A2‐G3‐K9‐TAT) showed hemolysis at low concentrations such as 25 µg mL^−1^,^[^ ^469^ ^]^ while PAMAM G4 dendrimer (G4) induced less than 5% hemolysis at a concentration of 1280 µg mL^−1^.^[^ ^474^ ^]^

### 7.3 Immunogenicity

The immunogenicity of NPs is well known.^[^ ^26^ ^]^ Judge et al. developed PEGylated liposomes and showed that the in vivo efficacy and safety of these systems could be severely compromised following repeated administration. This phenomenon was characterized by a loss of disease site targeting, enhanced clearance from the blood, and acute hypersensitivity. These outcomes were attributed to a surprisingly robust, long‐lived antibody response generated against PEG that resulted from the strong adjuvant effect of the payload (plasmid in this case).^[^ ^477^ ^]^ Therefore, investigators evaluated immunogenicity of NP formulations for drug delivery to the brain.^[^ ^84^, ^434^, ^478^ ^]^ Previous work showed that RVG‐surface decorated liposomes and exosomes slightly induced the release of pro‐inflammatory cytokines (TNF‐*α*, IFN‐ *γ*, IL‐1*β*, IL‐6) upon multiple injections to animal models.^[^ ^23^, ^478^ ^]^ However, PLGA and BSA NPs decorated with RVG or Tf did not present immunogenicity.^[^ ^434^ ^]^ Tf is a single chain 80 kDa protein that facilitates the movement of iron between the blood and brain. These NPs contained oxytocin with sizes in the range of 100–278 nm, with slight negative charges. The possibility of the NP formulations to induce an immunogenic reaction was assessed by measuring the amount of nitrite released by dendritic cells in the presence of NPs. Oxidation of nitric oxide yields nitrite, and nitric oxide release is an important marker for the innate immune response.^[^ ^479^ ^]^ Neither PLGA nor BSA NPs decorated with RVG produced significant amounts of nitric oxide. The same observation was made for PLGA and BSA NPs decorated with Tf. This study favored using RVG targeting ligands, as these NPs were half the size of Tf decorated NPs (RVG‐conjugated BSA (100.1 nm), Tf‐conjugated BSA (196.3 nm), Tf‐conjugated PLGA (191.7 nm), RVG‐conjugated PLGA (201.2 nm)).^[^ ^436^ ^]^ On the other hand, Arranz‐Gibert et al. found that the all L‐versions of THR (THRPPMWSPVWP) and HAI (HAIYPRH) peptides were immunogenic.^[^ ^480^ ^]^ The immunogenicity of these peptides were evaluated by several administrations of the peptide by i.p. injection into mice, and evaluation of the antibody in the blood by ELISA. It was demonstrated that two retro‐D‐peptides (H_2_N‐HRPYIAH‐CONH_2_ and H_2_N‐PWVPSWMPPRHT‐CONH_2_) were protease‐resistant and preserved the original BBB shuttle activity of the parent peptide, but were much less immunogenic than the parent peptides. As explained in the above, there are established in vivo and in vitro methods to evaluate the immunogenicity of NPs.^[^ ^22^ ^]^ In addition, Rudra et al. showed that using adoptive transfer experiments and T cell knockout models, it was possible to find antibody responses against self‐assembling peptides. Consequently, by deleting amino acid regions in the peptides recognized by T cells, immunogenicity was significantly diminished for self‐assembled nanofibers.^[^ ^481^ ^]^ This technique could be applied to identify effective brain targeting ligands with reduced immunogenicity.

Aptamers are small single stranded RNA or DNA molecules that bind to their target through shape recognition, similar to that of conventional antibodies. Aptamers have been developed as brain targeting ligands. Low immunogenicity is one of the main advantages of aptamers.^[^ ^482^ ^]^ For example, Pegaptanib sodium drug substance is a pegylated anti‐VEGF aptamer;^[^ ^483^ ^]^ for the treatment of choroidal neovascularization secondary to age‐related macular degeneration, which was well tolerated in clinical trials (phase II,^[^ ^484^ ^]^ phase III^[^ ^485^ ^]^) after multiple intravitreal injections. Although Macugen (pegaptanib) was withdrawn from the European market by the European Medicines Agency in 2011 (https://www.ema.europa.eu/en/documents/withdrawal‐report/withdrawal‐assessment‐report‐macugen_en.pdf), the decision was not based on immunogenicity of the product. In fact, the report states that only 4 patients out of 131 showed an IgG antibody response to non‐PEGylated pegaptanib following multiple injections over one year of the treatment. However, it should be noted that there are individuals positive for antinuclear antibodies. The report adds that 5/100 individuals were positive for antipegaptanib IgG, and 13/100 were positive for anti‐non‐PEGylated pegaptanib IgG, but the numbers do not appear significant.

The identification of aptamers is achieved typically by an in vitro selection process termed systematic evolution of ligands by exponential enrichment (SELEX).^[^ ^486^ ^]^ This method was applied to identify an aptamer that specifically targets the extracellular domain of the mouse TfR.^[^ ^487^ ^]^ Despite being a challenging process, aptamers are making their ways into neuroscience.^[^ ^488^, ^489^ ^]^ To make this process more efficient, Cheng et al. developed an in vivo evolution protocol to identify aptamers that home to the brain after injection to peripheral tissues. This method involved the injection of the aptamers intravenously, then harvesting mouse brain, extracting the aptamers, purification, amplification and re‐injecting into subsequent animals.^[^ ^490^ ^]^ The cycle was carried out for 22 rounds. Applying this method led to identifying an efficient aptamer with 71 nucleotides

(A15: 5′GGG AGG ACG AUG CGG CGU AUU GCG CGA GGA UUA UCC GCU CAU CGU UGU UGU UGU GCA GAC GAC UCG CCC GA3′) with the ability to target the brain. Although A15 showed enhanced accumulation in the brain, most of the i.v. injected aptamer was retained by the kidneys and liver. Although immunological reactions were not measured in this study, continuous testing of the aptamers in the mice suggests nonimmunogenicity of these ligands.

Monaco et al.^[^ ^491^ ^]^ conjugated the Gint4.T aptamer (5′UGU CGU GGG GCA UCG AGU AAA UGC AAU UCG ACA3′)^[^ ^492^ ^]^ to the surface of PLGA‐*b‐*PEG‐COOH NPs to yield PLGA‐*b*‐PEG‐Gint4 NPs with the size of 52 nm. The Gint4.T aptamer not only can cross the BBB, but also can target the *β* form of platelet‐derived growth factor receptors on glioblastoma cells.^[^ ^492^ ^]^ PLGA‐*b*‐PEG‐Gint4 appeared in the brain of mice bearing U87MG orthotopic xenografts following i.v. injection.^[^ ^491^ ^]^ Utilizing scrambled aptamer, the NPs did not accumulate in the glioblastoma.^[^ ^491^ ^]^ The immunogenicity of the NPs was not evaluated. In another study but with the same brain disease, Tang et al. labeled quantum dots with an aptamer (A32: 5′GCA ATG GTA CGG TAC TTC CTG AAT GTT GTT TTT TCT CTT TTC TAT AGT ACA AAA GTG CAC GCT ACT TTG CTA A3′), which binds to the epidermal growth factor receptor variant III (EGFRvIII) particularly expressed on the surface of glioma cells.^[^ ^493^ ^]^ These labeled quantum dots (QD‐Apt) accumulated in the glioblastoma cells in the brains of mice bearing U87‐EGFRvIII tumors. Although QD‐Apt NPs had diameters of about 20 nm, the accumulation of these NPs in the tumors was not because of the EPR. The QD‐Apt NPs did not induce inflammation in the brain, heart, liver, spleen, lungs, or kidneys after multiple injections over 28 days with 7‐day gaps between injections.^[^ ^493^ ^]^

In conclusion, aptamers are promising brain targeting ligands. This is because, the nonimmunogenicity profiles have been shown both in vivo and in clinical trials (Pegaptanib) for aptamers. While aptamers are susceptible to degradation by the nucleases in the serum, these may be overcome by altering the sugar position in the aptamer sequence and using oligonucleotides containing modified nucleotides in the aptamer sequence.^[^ ^494^ ^]^ More importantly, aptamers can self‐assemble into NPs with the sizes of 50 or 59 nm (depending on loaded drug), simply by mixing equimolar amounts of complementary oligonucleotides.^[^ ^495^ ^]^ Alternatively, aliphatic chains ((CH_2_CH_2_O)_24_)^[^ ^496^ ^]^ can be conjugated to aptamers to make amphiphilic aptamers. These aptamers can self‐assemble to NPs with size of 68 ± 13 nm,^[^ ^496^ ^]^ which makes them suitable for brain drug delivery. Interestingly, cholesterol‐conjugated aptamers self‐assembled to NPs of 120 nm,^[^ ^497^ ^]^ which is in agreement with the size of self‐assembled NPs (150 nm) from cholesterol‐TAT amphiphilic peptides.^[^ ^498^ ^]^ The latter NPs were able to penetrate the BBB and suppress bacterial growth in the brain. Therefore, cholesterol‐conjugated aptamer NPs may be able to deliver RNA interference to the brain.^[^ ^497^ ^]^ It should be noted that cholesterol‐TAT self‐assembled NPs induced hemolysis at concentrations as low as 50 µg mL^−1^,^[^ ^498^ ^]^ and this is in agreement with observations in our experiments for RVG based NPs (unpublished data). Aptamers, however, have not been reported to promote hemolysis. Hence, if brain targeting aptamers are employed in the self‐assembled NCs, then these may be employed as drug delivery to the brain with a better safety profile compared to other NP based drug delivery systems.

### 7.4 The Interaction between Microglia and Nanoparticles

Microglia are the resident immune cells of the brain, which are primarily involved in surveillance, phagocytosis, and production of cytokines such as Tumour necrosis alpha (TNF‐*α*) and trophic factors such as brain‐derived neurotrophic factor (an important role in neuronal survival and growth). Microglia respond rapidly to alterations in the brain homeostasis. Therefore, microglia can respond to the presence of NPs in the brain. The uptake of NPs by microglia can be as early as 3 h after the exposure of the cells to NPs.^[^ ^499^ ^]^ Activated microglia internalize more NPs compared to resting microglia.^[^ ^500^ ^]^ Polymeric NPs in the brain were internalized by microglia depending on the size and surface charge.^[^ ^501^ ^]^ In addition, gold NPs were internalized by microglia, which activated these cells.^[^ ^502^ ^]^ The surface chemistry of the NPs plays a major role in the activation of microglia.^[^ ^503^ ^]^ For example, surface coating of gold NPs with PEG did not activate microglia, while coating the NPs with cetyltrimethylammonium bromide activated microglia.^[^ ^503^ ^]^ Similarly, silver NPs (50 nm diameter) were internalized by microglia, and microglia dissolved these NPs within themselves. Interestingly, the silver NPs upregulated the expression of cystathionine‐*γ*‐lyase, which produces hydrogen sulfide (H_2_S) in the microglia. The increased levels of H_2_S reduced the release of TNF‐*α*, when the microglia were challenged with lipopolysaccharide.^[^ ^504^ ^]^ On the other hand, NPs can stimulate the microglia and as a result damage the neurons. These include SiO_2_‐NPs, TiO_2_‐NPs, Fe_3_O_4_‐NPs, and hydroxyapatite‐NPs. The interaction of these NPs with microglia released TNF‐*α*, IL‐1B, and IL‐6.^[^ ^505^ ^]^ Furthermore, neuroinflammation is associated with impaired microglia functions, and these cells have increased uptake of dendrimer NPs. This shows that in diseased brain the clearance of NPs by microglia could increase compared with healthy brain.^[^ ^506^ ^]^ As well as undesired uptake of NPs by microglia, NPs may be engineered to specifically target microglia. For example, curcumin‐loaded chitosan–bovine serum albumin NPs targeted microglia and activated these cells to accelerate the phagocytosis of A*β* peptide.^[^ ^507^ ^]^ As another example, Choi et al. showed that surface decoration of ceria–zirconia NPs (18 nm diameter) with microglia specific‐antibody (CD11b) allowed the uptake of these NPs by microglia and reducing the release of IL‐1*β* and IL‐6 due to the presence of reactive oxygen species.^[^ ^508^ ^]^ Similarly, inclusion of mannose in Pluronic‐F127 polymer and tannic acid (TA) based NPs increased the uptake of these NPs by polarized microglia. As polarized microglia overexpress mannose receptors.^[^ ^509^ ^]^

In conclusion, in the design of NPs for brain delivery, it should be noted that these NPs may not only be internalized by microglia, which leads to reduced efficacy of NPs, but also cause the activation of microglia, which could result in neuronal injuries. Therefore, NPs will be picked up by microglia, one way or another, this is their job. However, ingredients may be included in the NPs such that microglia will not be polarized to a pro‐inflammatory state.

## 8 Conclusion

This review article highlights changes in the BBB related to aging and diseases, and how these affect our ability to target the brain using NPs or NCs. Although aging and brain diseases increase the BBB permeability, the deposition of blood proteins in the BL can repair the BBB barrier functionality to some degrees. Aging changes the BBB morphology, permeability, and functionality. The expression of important receptors such as that for insulin or transporters such as P‐gp decreases. This is also applicable to the BBB in diseased states, with the receptor profile of the BBB changing. In AD the BBB permeability increases, however, the diffusion of NPs into the brain may be hindered due to A*β* deposition around brain microvessels. In MS, the BBB permeability increases, yet, NPs in the size of 20–40 nm find difficulties to cross the BBB in patients. The BBB integrity remains relatively intact in PD, hence, direct injections to the brain have been employed in clinical trials. On the other hand, the BBB permeability increases in brain tumors. Both targeted and nontargeted NPs in the size range of 3.25–400 nm have been employed in clinical trials, although the presence of brain targeting ligands improves drug delivery to the brain. Similarly, BBB permeability increases following ischemic stroke both in humans and animal models. Therefore, nontargeted NPs would cross the compromised BBB, while brain targeted NPs would have more efficacy. Administering NPs i.v. could result in complement activation, cytotoxicity and hemolysis, and induction of the release of pro‐inflammatory cytokines (TNF‐*α*, IFN‐ *γ*, IL‐1*β*, IL‐6). The activation of the complement system leads to the generation and deposition of opsonins such as C3b on the surface of the NPs (opsonization). The opsonized NPs are rapidly engulfed by macrophages, which leads to reduction of the nanoparticle therapeutic efficacy. NPs could cause death, apoptosis or necrosis of neurons. This is important when NPs or NCs are employed to deliver noncytotoxic therapeutic agents to the brain such as nerve growth factor. Brain targeting NPs could cause hemolysis, in particular amphiphilic peptides. If hemolysis is significant, then this could lead to anemia. Furthermore, the rupture of red blood cells may affect NP opsonization, and influence distribution and delivery to the intended target sites, or promote uptake and clearance by macrophages. In vitro BBB models aim to inform the potential for pharmaceutical drug candidates to cross the BBB and reach the neurons or brain extracellular environment. Although current in vitro methods are far from a perfect facsimile of the BBB in vivo, good correlation between in vitro and in vivo drug permeability has been reported.

We have described the biological and physical background to many of these current challenges and opportunities for the development of new therapeutic options to treat diseased and damaged brains. Navigating the opportunities provided by physiological changes in the BBB and exploiting the latest models must be done while simultaneously avoiding the challenges of complement activation, immunogenicity, cytotoxicity and other toxicity. The prize for navigating this path could be a revolution in therapies targeting the brain.

## 9 Future Directions

The physiological and pathological changes in the BBB should be taken into account for drug delivery to the brain using NPs. This encompasses changes in the receptor profile of the BBB and modifications in the BL, which makes diffusion of NPs difficult in the brain parenchyma. Therefore, brain targeting ligands should be carefully selected to ensure efficient delivery of the NPs across the BBB. It is possible that enzyme responsive NPs would provide the benefit of releasing the drug content in the brain parenchyma facilitating fast diffusion of small active therapeutic agents in the brain parenchyma. Furthermore, enzyme responsive NPs may prevent activation of brain‐resident macrophages, because rapid disintegration before recognition by microglia. Certainly, noninvasive brain‐targeting NPs would be the way forward. This is not only to minimize further damages to the diseased brain, but also to reduce the costs of treatment by eliminating the need for a surgeon to make a hole in the skull for injecting the active ingredient/NPs into the brain. The scalability and reproducibility of NP production should be taken into consideration. Simpler procedures would not only reduce the complexity of the manufacturing process of NPs, but also improve batch‐to‐batch consistency, which is favored by regulatory authorities. Certainly, there is a requirement for the development of safe and nonthrombogenic NPs that target the blood clots in the brain vessels that underlie the brain ischemic stroke. Rapid responding NPs will be favored to re‐establish the blood flow in the brain as quickly as possible following the stroke. In this regard, enzyme‐inhibiting NPs would also be useful during the early hours of the stroke. This is because, following an ischemic stroke, the levels of degenerative enzymes such as MMP‐9 increase in the brain, which are correlated with poor disability outcomes.

## Conflict of Interest

The authors declare no conflict of interest.
